# Opposite Phenotypes of Muscle Strength and Locomotor Function in Mouse Models of Partial Trisomy and Monosomy 21 for the Proximal *Hspa13-App* Region

**DOI:** 10.1371/journal.pgen.1005062

**Published:** 2015-03-24

**Authors:** Véronique Brault, Arnaud Duchon, Caroline Romestaing, Ignasi Sahun, Stéphanie Pothion, Mona Karout, Christelle Borel, Doulaye Dembele, Jean-Charles Bizot, Nadia Messaddeq, Andrew J. Sharp, Damien Roussel, Stylianos E Antonarakis, Mara Dierssen, Yann Hérault

**Affiliations:** 1 Institut de Génétique et de Biologie Moléculaire et Cellulaire, Illkirch, France; 2 Centre National de la Recherche Scientifique, UMR7104, Illkirch, France; 3 Institut National de la Santé et de la Recherche Médicale, U964, Illkirch, France; 4 Université de Strasbourg, Illkirch, France; 5 LEHNA, CNRS UMR502, Université de Lyon, Villeurbanne, France; 6 Genes and Disease Program, Center for Genomic Regulation, Barcelona, Spain, and CIBER de Enfermedades Raras (CIBERER), Barcelona, Spain; 7 Transgenese et Archivage Animaux Modèles, TAAM, CNRS, UPS44, Orléans, France; 8 Department of Genetic Medicine and Development, University of Geneva Medical School, Geneva, Switzerland; 9 Key-Obs S.A., Allée du Titane, Orléans, France; 10 Department of Genetics and Genomic Sciences, Mount Sinai School of Medicine, New York, New York, United States of America; 11 iGE3 Institute of Genetics and Genomics of Geneva, Geneva, Switzerland; 12 Institut Clinique de la Souris, PHENOMIN, GIE CERBM, Illkirch, France; Stanford University School of Medicine, United States

## Abstract

The trisomy of human chromosome 21 (Hsa21), which causes Down syndrome (DS), is the most common viable human aneuploidy. In contrast to trisomy, the complete monosomy (M21) of Hsa21 is lethal, and only partial monosomy or mosaic monosomy of Hsa21 is seen. Both conditions lead to variable physiological abnormalities with constant intellectual disability, locomotor deficits, and altered muscle tone. To search for dosage-sensitive genes involved in DS and M21 phenotypes, we created two new mouse models: the Ts3Yah carrying a tandem duplication and the Ms3Yah carrying a deletion of the *Hspa13-App* interval syntenic with 21q11.2-q21.3. Here we report that the trisomy and the monosomy of this region alter locomotion, muscle strength, mass, and energetic balance. The expression profiling of skeletal muscles revealed global changes in the regulation of genes implicated in energetic metabolism, mitochondrial activity, and biogenesis. These genes are downregulated in Ts3Yah mice and upregulated in Ms3Yah mice. The shift in skeletal muscle metabolism correlates with a change in mitochondrial proliferation without an alteration in the respiratory function. However, the reactive oxygen species (ROS) production from mitochondrial complex I decreased in Ms3Yah mice, while the membrane permeability of Ts3Yah mitochondria slightly increased. Thus, we demonstrated how the *Hspa13-App* interval controls metabolic and mitochondrial phenotypes in muscles certainly as a consequence of change in dose of *Gabpa*, *Nrip1*, and *Atp5j*. Our results indicate that the copy number variation in the *Hspa13-App* region has a peripheral impact on locomotor activity by altering muscle function.

## Author Summary

Down syndrome is the most common genetic cause of intellectual disabilities, and marked hypotonia is among the constant diagnostic traits. Here we observe the opposite changes in locomotion, muscle strength, and energetic balance in new mouse models of DS and M21 for the *Hspa13*-*App* proximal region of human chromosome 21. The differential expression analysis revealed downregulation of skeletal muscle genes controlling energetic metabolism, mitochondrial activity, and biogenesis in Ts3Yah, while upregulation of similar set of genes was found in Ms3Yah mice. This phenomenon correlates with the changes in mitochondrial proliferation with increased membrane permeability of Ts3Yah mitochondria and decreased mitochondrial ROS production in Ms3Yah mice. Our results demonstrate the opposite phenotypic effect of trisomy and monosomy of the *Hspa13-App* syntenic region of human chromosome 21, highlighting new physiological mechanisms for hypotonia in DS individuals.

## Introduction

DS and M21 are complex genetic conditions that arise from an altered dosage of genes on human chromosome 21 (Hsa21). Full M21 is rare and generally not compatible with life, with only a few cases reported in literature, and the oldest case survived for only a few days after birth [1–5]. The remaining M21 cases are partial deletion or mosaics. The phenotypes of M21 are variable among individuals and depend on the specific deleted region of Hsa21. Some patients have only mild to moderate intellectual disabilities [6, 7], while others are more severely affected and present multiple dysmorphic craniofacial, skeletal, and cardiac features, as well as muscular, ocular, pulmonary, renal, and genitourinary abnormalities [8–12].

DS, with an incidence of one in 700 live births [13], is still the most common viable cause of chromosomal aneuploidy in humans and the most frequent genetic cause of intellectual disability. Besides the characteristic of facial dysmorphology, hypotonia is one of the major traits observable at birth in this disorder. More than 80 features occur with various degrees of expression and frequency in DS, and some have been associated with regions of Hsa21 [12, 14]. However, the causes of delayed motor performance affecting both gross and fine motor skills, weak muscle strength, and exercise performance are not known. Motor impairments are prominent throughout life and may contribute to the delay in cognitive skills [15, 16]. Poor locomotor skills have been attributed to impaired coordinated input due to cerebellar dysfunction [17, 18] but might have a more complex origin combining deficit in balance and postural control, stiffness, reduced muscular strength, and hypotonia.

Hypotonia is a state of low muscular tonicity due to decreased springlike properties of the striated muscle fibres, which suffer from a lack of energy to keep working at a normal level for a long time and a slower speed of response. Hypotonia may also be accompanied by reduced muscular strength. It can be caused by abnormal muscle function, abnormal neurological input, or metabolic diseases. Impaired exercise endurance is observed in DS individuals and could arise from endocrinal or metabolic perturbations [19, 20]. However, little is known about the origin of hypotonia in DS.

Mouse models provide a powerful tool to study the physiological, cellular, and molecular aspects of human diseases. Moreover, the possibility to manipulate large genomic regions in the mouse genome has offered a chance to develop mouse models of aneuploidies and investigate the relationship between phenotype and genotype. Hsa21 genes are found in three syntenic regions localised on mouse chromosomes 16 (Mmu16, 23.3 Mb, 115 Hsa21 orthologous genes), 17 (Mmu17, 1.1 Mb, 19 Hsa21 orthologous genes), and 10 (Mmu10, 2.3 Mb, 41 Hsa21 orthologous genes) (http://www.ensembl.org).

Several mouse models have been generated over the years that display many features of DS [21–25], but locomotor activity has not previously been fully investigated in these models. There is little in literature about the locomotor phenotype of the trisomic models for genes located on the Mmu16. Moreover, conflicting results have been reported on the motor abilities of Ts65Dn mice that are trisomic for 102 orthologous of Hsa21 genes (http://www.ensembl.org) located between *Mprl39* and *Zfp295* [26, 27]. Additionally, this model is trisomic for 50 genes and 10 pseudogenes present in the centromeric part of Mmu17 that is irrelevant to DS [28]. Studies from Costa and collaborators reported lower maximal treadmill running speeds, deficits in balance and motor coordination assessed by the rotarod test, and reduction in grip force [29, 30], whereas Hyde and collaborators found no impairment in motor learning in Ts65Dn mice [31, 32]. Several analyses have described abnormal electrical and biochemical properties in motor neurons and muscle cells both in human and DS mouse models [33–35]. The analysis of the distal part of the Hsa21 did not reveal locomotor deficit in a mouse model trisomic for the *Abcg1-U2af1* segment on Mmu17 [25] and demonstrated that the *Cstb-Prmt2* segment is also not necessary, as going back to two copies of this region in the Tc1 transchromosomic model does not rescue the locomotor deficit observed in this model (although five genes in this region were not tested here as they are not trisomic in Tc1) [36].

To assess the contribution of the DS phenotype associated with the 9.4 Mb *Hspa13-App* Hsa21 syntenic region on Mmu16 that is not trisomic in the widely used Ts65Dn model, we generated through chromosome engineering a trisomic mouse model (Ts3Yah) and the corresponding monosomic model (Ms3Yah) for this region. Those mice were evaluated for different behavioural phenotypes associated with DS and displayed opposite locomotor phenotypes. While Ts3Yah mice have impaired locomotion that is associated with increased grip strength and muscle mass, the opposite phenotype was observed in Ms3Yah animals. The transcription profile analysis of skeletal muscles revealed coordinate changes in muscle metabolic activity and mitochondrial biogenesis. This finding was confirmed by alteration in the oxidative capacity and mitochondrial content of the skeletal muscles. This study unravels new insight into the metabolic changes that result in defects of skeletal muscle strength and performance that are associated with Hsa21 aneuploidy and suggest new candidate genes involved in DS and M21.

## Results

### Generating models of aneuploidy for the *Hspa13-App* region on mouse chromosome 16

**Fig 1:**
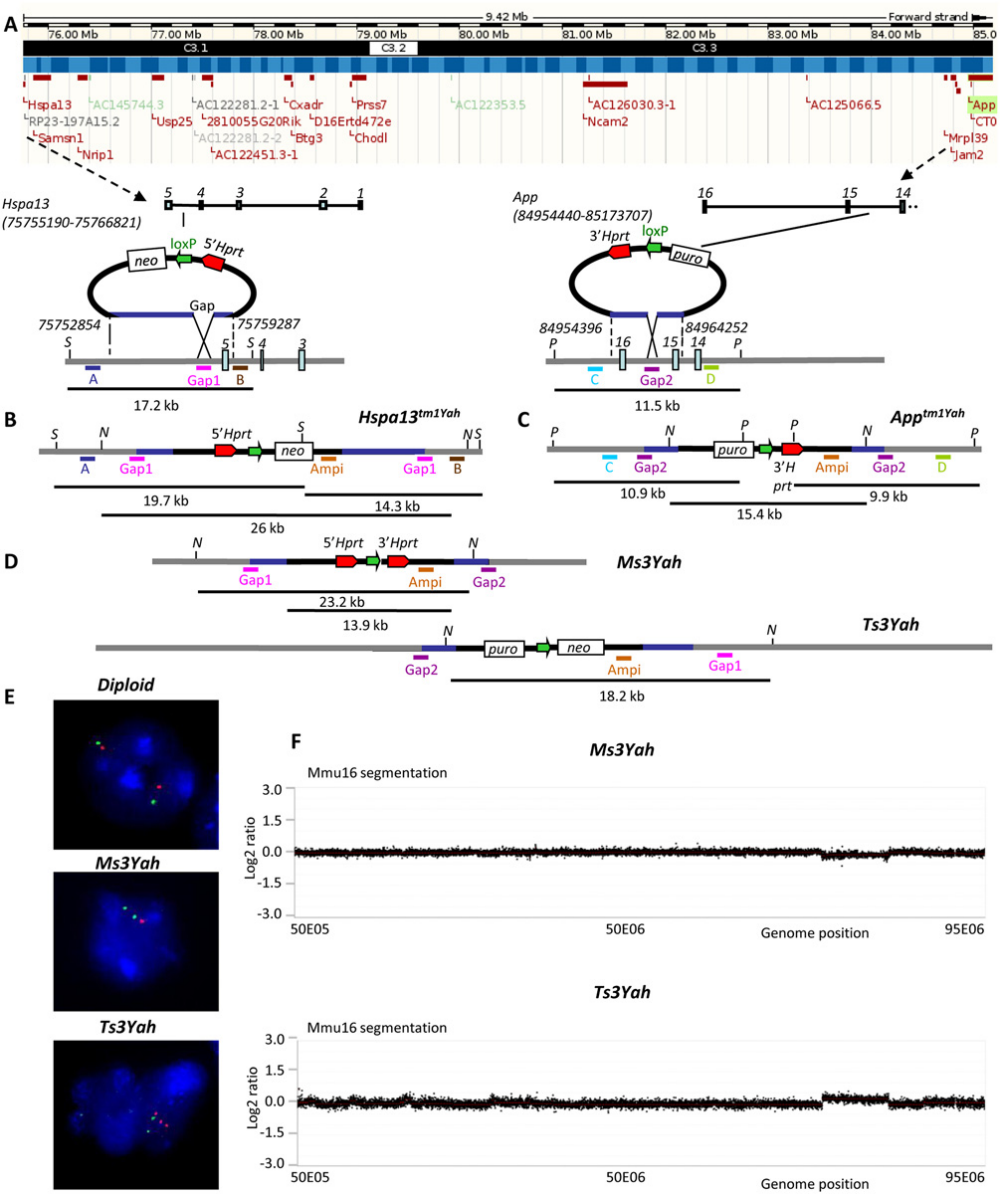
Generating a 9.2 Mb deletion and reciprocal tandem duplication between the *Hspa13* and *App* loci. (A) The 9.2 Mb targeted region defined by the *Hspa13* and *App* genes as shown from a capture derived from the UCSC genome browser (http://genome.ucsc.edu/). Black headband: chromosomes bands; blue headband: contigs; red rectangle boxes: protein coding genes. (B) The targeting vectors containing a loxP site (green arrow), a selectable antibiotic resistance gene (puro or neo), and a part of the *Hprt* gene (3’ or 5’ *Hprt*, red arrows) were integrated successively in the *Hspa13* locus (Hspa13tm1Yah) and in the *App* gene (Apptm1Yah). The start and end positions of the genomic homologous regions present in the 5’ and 3’-Hprt vectors are from mouse genome assembly GRm38. The *Stchtm1Yah* allele was checked by Southern blot analysis. The digestion of DNA with *SacI* (S) restriction enzyme and hybridisation with external probes A and B gives additional fragments of 19.7 kb and 14.3 kb, respectively, for the *Stchtm1Yah* allele compared to the wild-type (wt) allele of 17.2 kb. The hybridisation of the same DNA digest with the *Gap1* probe gives two additional fragments of 19.7 and 14.3 kb for the *Hspa13tm1Yah*. (C) The integration of the 3’*Hprt* vector was confirmed in the same manner by digestion with the *Psi*I (P) enzyme and hybridisation with probes C (wt, 11.5 kb; Apptm1Yah allele, 10.9 kb), D (wt, 11.5 kb; *Apptm1Yah* allele, 9.9 kb), and Gap2 (wt, 11.5 kb; *Apptm1Yah* allele, 10.9 and 9.9 kb). (D) After Cre-mediated recombination, the deletion (Ms3Yah allele) and duplication (Ts3Yah allele) were identified by *Nde*I (N) digestion, and a probe was made on the ampicillin gene (*Ampi*) present in both targeting vectors that recognises recombinant clones with the Ms3Yah (23.2 kb) and Ts3Yah (18.2 kb) alleles from nonrecombinant ones that still carry the *Hspa13tm1Yah* (26 kb) and *Apptm1Yah* (15.4 kb) alleles. Puro: puromycin, neo: neomycin. (E) Interphase FISH analysis with BAC probes that map in the region of the deletion or duplication (BAC bMQ-34K13; red) and outside (BAC bMQ-381L17; green). The wild-type (diploid) showed two red and two green adjacent signals. On the other hand, nuclei from Ms3Yah showed two green and only one red signal due to the deletion of the *Hspa13-App* region, and nuclei from Ts3Yah showed two green and three red signals. (F) The CGH profile of Mmu16 was established using NimbleGen mouse HD2 whole genome CGH oligonucleotide arrays comprising 2,100,000 isothermal probes 50–75 bp length, with a median spacing of 1.1 kb throughout the genome (UCSC NCBI37/mm9/July 2007 assembly) enabling high-resolution CNV detection. It confirmed the loss of one copy of the *Hspa13-App* fragment in Ms3Yah mice and the presence of an additional copy of the same fragment in Ts3Yah animals. Plotted are log 2 transformed hybridisation ratios of Ms3Yah and Ts3Yah versus diploid mouse DNA.

Chromosomal engineering based on the MICER [37] and long-range Cre/loxP-mediated recombination [38] were used in embryonic stem (ES) cells to produce the segmental duplication (Dp[16*Hspa13-App*]2Yah; MGI5519056) and the corresponding deletion (Del[16*Hspa13-App*]3Yah; MGI5519049) of the *Hspa13-App* 9.4 Mb interval located on Mmu16, as described in Materials and Methods and Fig. 1. This mouse syntenic region contains 15 Hsa21 orthologous genes (Fig. 1A, S1 Table). As a result of the insertion points of the targeting vectors, the *Hspa13* gene at the proximal end of the 9.5 Mb region was in two functional copies in the duplication and lost in the deletion, whereas the *App* gene at the distal end was only in one functional copy in the duplication and lost in the deletion. The derived aneuploid mouse models corresponding to the tandem duplication and the reciprocal deletion were respectively named Dp(16)(*Hspa13*-*App*)2Yah for the trisomy (abbreviated as Ts3Yah) and Del(16)(*Hspa13*-*App*)3Yah for the monosomy (abbreviated as Ms3Yah). The two models were validated by the Southern blot analysis (Fig. 1B), fluorescent *in situ* hybridisation (FISH) analysis (Fig. 1E), and array-based comparative genomic hybridisation (Fig. 1F) as described in Materials and Methods. Ts3Yah and Ms3Yah mice are viable and fertile, with normal life spans, and exhibit no obvious gross anatomical anomalies on the C57BL/6J genetic background. Ts3Yah show no weight difference (weight at 10 weeks, n = 12 male mice per genotype: control 26.4±0.9g vs Ts3Yah 26.5±0.7g, Student’s t-test *p* = 0.97). While their weight is not different at birth from their littermates (weight taken at J2, n = 9 male mice per genotype: control 1.8g±0.1g vs Ms3Yah 1.9g±0.1 g, Student’s t-test *p* = 0.78), adult Ms3Yah mice weight approximately 10% less than their wild-type littermates (weight at 10 weeks, n = 10 male mice per genotype: control 28.6±0.6 g vs Ms3Yah 24.6g±1.1g, Student’s t-test *p* = 0.005).

### Locomotor performances are oppositely affected in mice trisomic and monosomic for the *Hspa13-App* region

**Fig 2:**
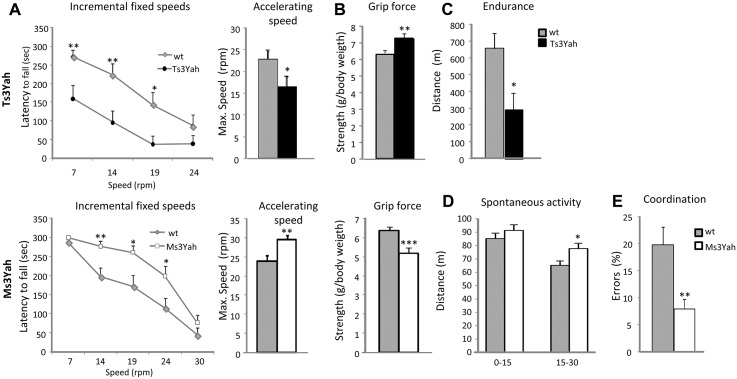
Evaluation of motor skills. (A) The motor skill performances of Ts3Yah mice, Ms3Yah mice, and their diploid controls (n = 10–15 per genotype) were analysed using two rotarod tests. The first test consisted of four consecutive trials of 5 minutes at constant speed and reported as the mean ± sem of the time that mice remained on the rod (repeated ANOVA ‘genotype,’ ‘speed’: Ts3Yah vs diploid: F[1, 69] = 10.042 *p* = 0.004; Ms3Yah vs diploid: F[1, 87] = 8.809 *p* = 0.006). This test was followed by another test involving two trials at accelerating speed (4–40 rpm) for 5 minutes and the calculated mean rotational velocity at the time of falling for the two trials and each group. (B) Grip force, immediately prior to the animal releasing its grasp from the grid, was measured in grams and normalised to the weight of the animal. (C) The endurance of Ts3Yah and Ms3Yah mice to exercise was measured as the maximal running distance travelled on a treadmill until exhaustion. (D) Spontaneous locomotor activity and habituation were assessed in an open field by measuring the distance travelled during the first 15 minutes and the last 15 minutes of a 30-minute session. (E) Motor coordination skills were tested with a notched bar by looking at the percentage of errors made by the mouse with its hind paws when crossing the notched bar. Data are presented as mean ± sem and analysed with Student’s t-test, *p<0.05, **p<0.01, ***p<0.001.

Motor function was assessed first using two rotarod motor performance tests: one with five-minute sessions of incremental fixed speeds and the second consisting of two five-minute trials with accelerating speed. Ts3Yah mice showed performances that were significantly lower than their diploid littermates in both tests at age 8 to 12 months, and Ms3Yah mice performed significantly lower than their diploid littermates at the same age as well (Fig. 2A). Looking at both Ts3Yah and Ms3Yah mice, the weight of the animal was not correlated with its performance, excluding that the increased performance in Ms3Yah animals was due to mice being leaner. The effects of the trisomy and monosomy on rotarod test performance were however not observed in the young mice aged 3 months (S1A Fig). In order to assess muscle strength, the maximal grip force was measured and normalised to the body weight of the animal. Muscle strength was increased in Ts3Yah and decreased in Ms3Yah mice compared to diploid littermates at 3 to 4 months and 8 to 12 months of age (Fig. 2B, at 8 to 12 months of age). Muscle endurance capacity was tested by having the mice run on a treadmill and measuring the maximal distance travelled. Ts3Yah mice were only able to run about half of the distance covered by diploid control mice, indicating that they were more susceptible to fatigue (Fig. 2C), whereas Ms3Yah mice showed performances comparable to diploid controls (S1B Fig). Spontaneous locomotor activity and habituation were measured in an open field by recording the distance travelled by the mouse during the first 15 minutes and the last 15 minutes of a 30 minute session. No difference was found in Ts3Yah mice (S1C Fig), while Ms3Yah mice had a slight overall increase in the distance travelled and did not reduce their exploratory activity as much as the controls animals during the last fifteen minutes of the session, suggesting a slight hyperactivity (Fig. 2D). The mice were tested for hind limb coordination by walking across a notched bar. No significant difference was observed for the percentage of errors in hind limb coordination in Ts3Yah mice (S1D Fig), while Ms3Yah mice made significantly less errors than their control littermates (Fig. 2E).

### Copy number variation of the *Hspa13-App* region triggers opposite expression changes of pathways involved in mitochondrial function and biogenesis in the muscle

**Fig 3:**
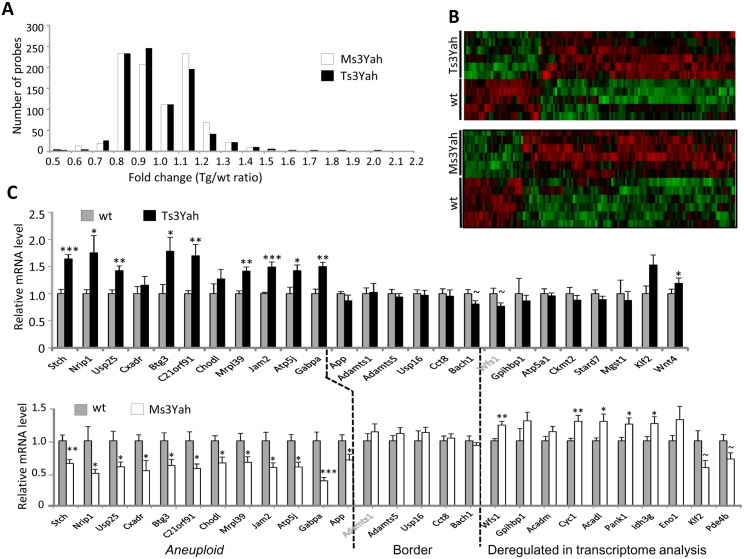
Gene expression profile of skeletal muscles from Ts3Yah and Ms3Yah transgenic mice. (A) Distribution of the deregulated genes depending on their fold change. (B) Clustering derived from statistically deregulated genes (Student’s t-test, *p*<0.05) with FC >|1.2| in Ts3Yah compared to their diploid control littermates and in Ms3Yah compared to their diploid control littermates. The red and green colours indicate relative expression levels (red: increased expression; green: decreased expression). (C) Quantitative PCR analysis of genes within the *Hspa13-App* trisomic or monosomic region (aneuploid), bordering the *Hspa13-App* region and selected for their significant deregulation within the transcriptome. Data are presented as mean ± sem and analysed with Student’s t-test, ~*p*<0.1, *p<0.05, **p<0.01.

Muscle strength and locomotion variation suggest that the observed motor phenotypes at least partly result from a peripheral muscular phenotype. Hence, we decided to focus our attention on the skeletal muscles. To identify molecular changes in the skeletal muscles of Ts3Yah and Ms3Yah mice, we studied the transcriptional profiles of gastrocnemius muscles isolated from adult mice. From the 45,281 probe sets sampled on the microarray, 43,058 and 43,632 were expressed in the Ts3Yah and the Ms3Yah, respectively. There were 2,302 (corresponding to 1,614 genes) and 2,246 (corresponding to 1,631 genes) probes differentially expressed in Ts3Yah and Ms3Yah muscles, respectively, compared to their euploid littermate controls, with a significant *p*-value <0.05 and resampling *p*-values of 0.081 (Ts3Yah) and 0.073 (Ms3Yah) (see Materials and Methods for details of the methods used). About half of them (1,129 and 1,169, respectively) were upregulated and half of them (1,173 and 1,077, respectively) downregulated. Fold changes (FC) observed varied between 0.5 and 2.1 for both Ts3Yah and Ms3Yah, with only about 5% of the deregulated genes having a FC>1.2 or <0.8 (Fig. 3A), indicating that the trisomy and the monosomy of the *Hspa13-App* region do not have a dramatic effect on gene expression. The final lists of the most deregulated probes (FC>|1.2|) for Ts3Yah and Ms3Yah are presented along with their annotation, FC, and *p*-value in Supplementary Tables (S2–S3 Tables), respectively, and the clustering of those deregulated genes are presented in Fig. 3B. Three genes from the *Hspa13-App* region (*Chodl*, *Atp5j*, and *D16Ertd472e [C21orf91 in human])* were among the most deregulated in Ts3Yah muscles. In addition, several protein kinases (*Ttn*, *Nmrk2*, *Csnk2a1-rs3*, *Pctk1*, *Pacsin3*, *Prkaa2 Raf1*, *Cdc2l2*, *Smg1*, *Hk2*, *Ckmt2*, *Dapk2*, *Mpp3*, and *Atp1b1*), three members of translation initiation factors (*Eif2c2*, *Eif4e3*, and *Eif2b5*), and a binding protein of a translation initiation factor (*Eif4ebp2*) were overexpressed, and a few mitochondrial-located proteins and enzymes were underexpressed (*Atp5a1*, *Ckmt2*, *Mgst1*, *Stard7*, *Abhd10*, *Cyb5b*, *Pdpr*, *Hk2*, *Tmem65*, *Tomm22*, *Hsd3b2*, and *Tmem160*). In Ms3Yah muscles, only 26 genes were downregulated at FC<0.8, and six of them (*App*, *Atp5j*, *Chodl*, *D16Ertd472e*, *Usp25*, and *Jam2*) corresponded to genes mapping within the *Hspa13-App* monosomic segment. Interestingly, among the 84 genes upregulated in Ms3Yah muscles, 29 were mitochondrial related (*Hadhb*, *Alas1*, *Ndufa8*, *Brp17*, *Aifm1*, *Acadl*, *Gbas*, *Mfn2*, *Acaa2*, *As3mt*, *Mrpl47*, *Adh1*, *Tmem143*, *Akr1b10*, *Slc25a11*, *Pptc7*, *Cyc1*, *Uqcc*, *LOC433224*, *Slc25a20*, *Mpc2*, *Rmnd1*, *Coq5*, *Slc25a3*, *Slc25a4*, *Idh3g*, *Hmgcl*, *Akr1b10*, *Coq6*, *Ppif*, and *Ndufaf4*). By comparing the lists of the most deregulated probes in Ts3Yah and Ms3Yah muscles, we found eight different genes displaying opposite deregulation in the two conditions: *Chodl*, *Atp5j*, and *D16Ertd472e* in the *Hspa13-App* region and *Wfs1*, *Ffar3*, *mt-Nd4l*, *Gpihbp1*, and *Hist1h4h* located elsewhere (see genes in bold in S2–S3 Tables).

We selected the genes located in the *Hspa13-App* interval and at its borders for the analysis of their expression profile by quantitative PCR analysis (QRT-PCR) (Fig. 3C). Only three genes within the *Hspa13-App* interval, namely, *Ncam2*, *Samsn1*, and *Prss7 (also known as Tmprss15)*, were not detected by QRT-PCR analysis in the striated muscle as reported in the literature. Nevertheless, most of the genes present within this interval and were not detected by the microarray experiment were found expressed in skeletal muscles by QRT-PCR analysis. The genes present in three copies in Ts3Yah muscles were upregulated at around 1.5 fold, at the exception of *Cxadr* and *Chodl*. As expected, the expression of *App* that is not duplicated in the Ts3Yah chromosome was unchanged in Ts3Yah muscle. In Ms3Yah animals, all the monosomic genes were globally 0.5 fold underexpressed compared to their disomic controls. In addition, we measured the expression levels of five genes at the border of the rearrangement, namely, *Adamts1*, *Adamts5*, *Usp16*, *Cct8*, and *Bach1* (Fig. 3C). No significant change of expression was observed for border genes present in two copies in the genome of Ts3Yah and Ms3Yah mice. Finally, genes found differentially expressed in Ts3Yah and Ms3Yah were tested by QRT-PCR. Most of the genes were validated and found to be significantly deregulated in Ms3Yah muscle as in the microarray analysis, although deregulation was only observed for *Wnt4* in Ts3Yah animals (Fig. 3C).

**Fig 4:**
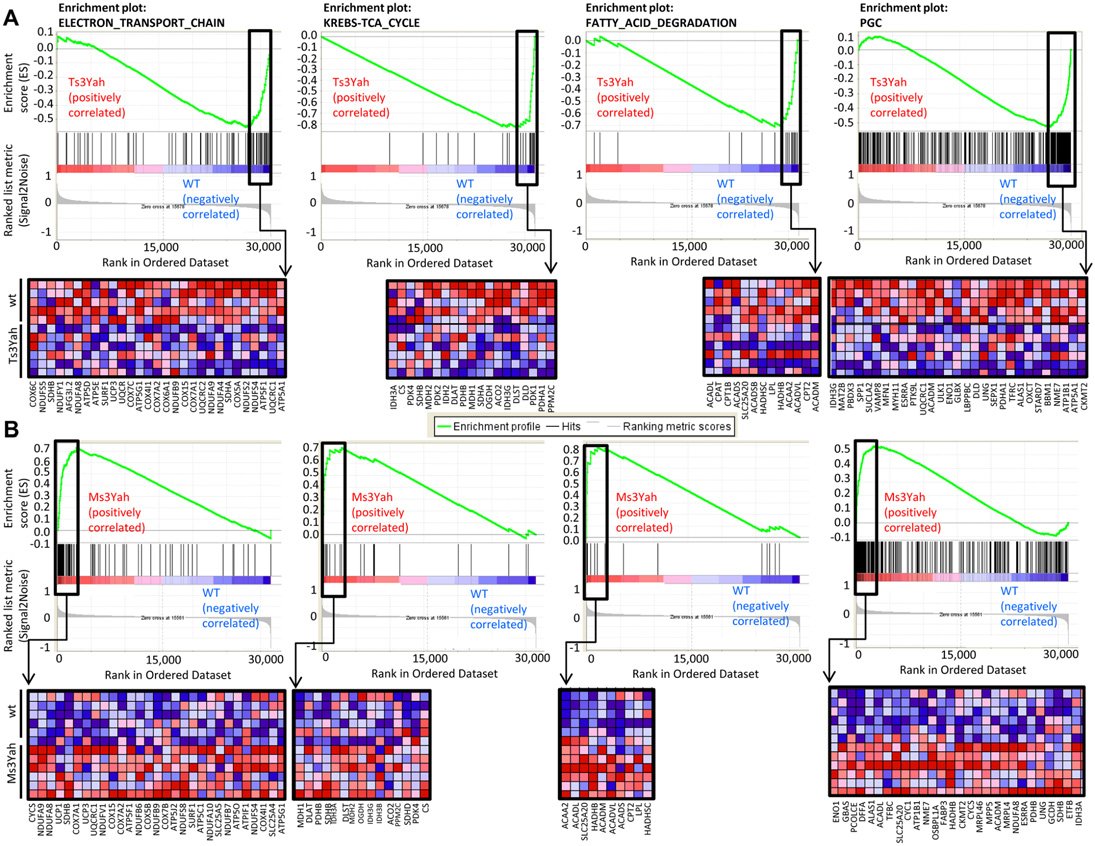
Pathways related to energy metabolism are underexpressed in Ts3Yah skeletal muscles and overexpressed in Ms3Yah skeletal muscles. GSEA-scoring plots of gene expression profile in gastrocnemius muscles from Ts3Yah (A) and Ms3Yah (B) mice. Four plots of representative gene sets relevant to metabolic pathways that were significantly enriched and the ‘PGC’ gene set corresponding to genes involved in OXPHOS that were activated by PGC1-α are shown. The top part of each plot shows the running enrichment score for the gene set as the analysis goes down the ranked list. The middle portion shows the position of members of the gene set in the ranked list of genes. The lower portion plots the value of the ranking metric of the genes in the expression data set. Below the enrichment plots, the corresponding heat maps show the leading edge subsets of genes that contribute most to the enrichment score. The red and blue colours indicate relative expression levels (red: increased expression; blue: decreased expression).

We further analysed our microarray data with the software tool ‘Gene Set Enrichment Analysis’ (GSEA; http://www.broad.mit.edu/gsea) [39, 40]. This analysis helps find correlation of gene expression. The genes belonging to a defined pathway are ranked together according to their change in expression in Ts3Yah and Ms3Yah muscles compared to their diploid controls and calculating a maximum enrichment score (ES) for each gene set. More than 20 informative gene sets were significantly downregulated in Ts3Yah and upregulated in Ms3Yah muscles compared to diploid muscles (S4–S5 Tables), whereas no category was found for upregulated in Ts3Yah or downregulated in Ms3Yah muscles. Most of the deregulated gene sets were associated with mitochondrial energy metabolism (electron transport chain and oxidative phosphorylation or ‘VOXPHOS’), fatty acid metabolism (fatty acid degradation), and carbohydrate metabolism (TCA cycle, glycolysis and gluconeogenesis, and butanoate metabolism). About half of the genes assigned to mitochondrial gene sets overlapped with those categories. The complementary GSEA ([40]) revealed a major overlap in the leading edge subset of genes (genes in the gene set that contribute most to the ES) between Ts3Yah and Ms3Yah enriched gene sets (see S4–S5 Tables for leading edge genes in each gene set) (see among the top-ranked genes sets, one called ‘PGC,’ for peroxisome proliferator-activated receptor-gamma coactivator) and corresponding to a set of genes involved in oxidative phosphorylation and whose expression is coordinately decreased in human diabetic muscles and activated upon expression of *Pgc-1α* (*Ppargc-1α*) in C2C12 cells [41]. Leading edge genes from this set show a 25% to 50% overlap with leading edge genes from genes sets implicated in energy and metabolic pathways identified in our analysis. Fig. 4 shows GSEA-scoring plots and their corresponding heat maps for ‘PGC’ and the four gene sets, including ‘electron transport chain,’ ‘TCA cycle,’ ‘fatty acid degradation,’ and ‘glycolysis/gluconeogenesis,’ that show the most overlap with gene set ‘PGC.’ Together, these findings suggest a shift in energy metabolism to a more oxidative state with increased mitochondrial biogenesis and/or activity in Ms3Yah skeletal muscles and an opposite suppression of the oxidative state in Ts3Yah muscles that might be triggered by signaling pathway(s) involving transcriptional coactivators of the PGC family or other cooperating partners involved in this regulatory pathway.

### The *Hspa13-App* region is implicated in the regulation of skeletal muscle oxidative capacity

**Fig 5:**
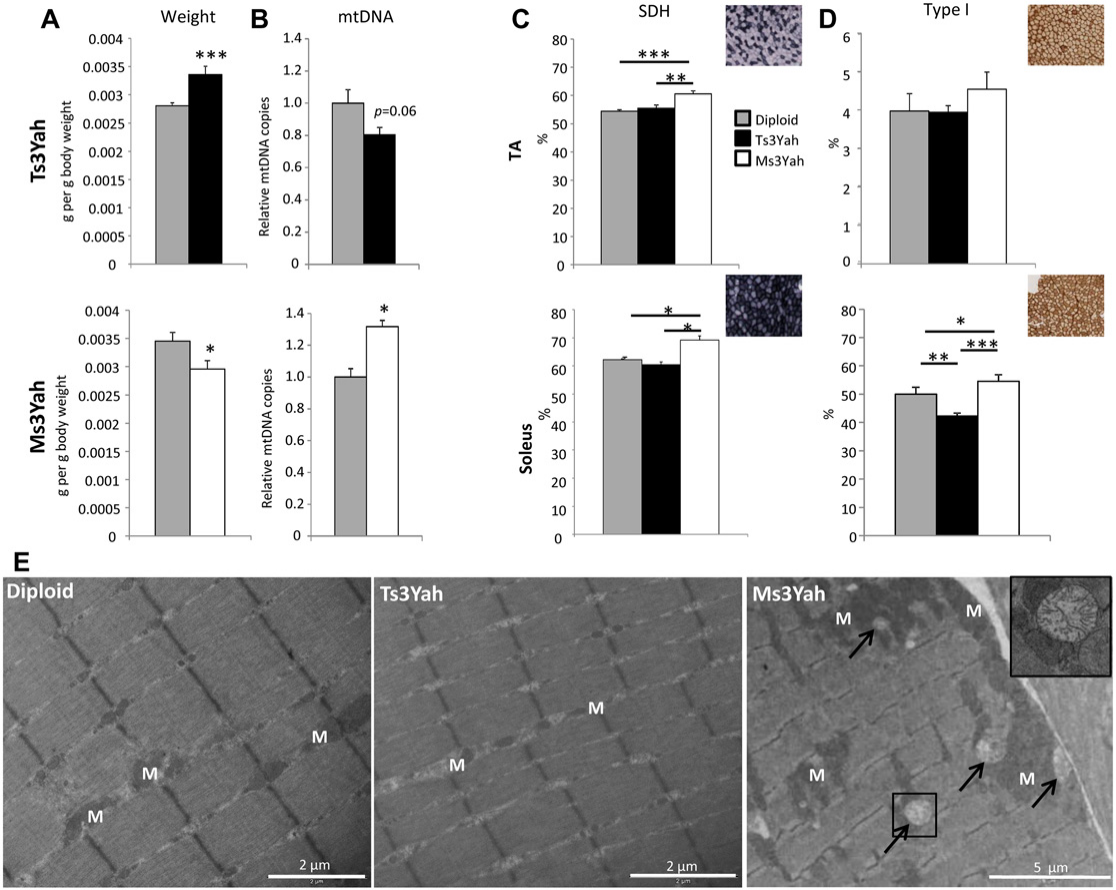
Histological analyses of skeletal muscles. (A) Mass of gastrocnemius muscles reported to body weight of the mice (n = 10–15 per group). (B) Quantification of the mitochondrial copy number in the gastrocnemius muscles of Ts3Yah and Ms3Yah mice relative to their diploid controls. The levels of mtDNA were normalised to levels of nucleus-encoded DNA (n = 5 per group). (C) Transverse sections of TA and soleus muscle were stained for SDH activity and SDH+ fibres counted in the entire sections. Results are given as percentages of stained fibres (n = 5–7 per group). (D) Changes in the proportion of type I slow-twitch fibres in TA and soleus of Ts3Yah and Ms3Yah mice using MyHC immunohistological staining (n = 5–7 per group). Data are presented as mean ± sem. Student’s t-test (A and B) and ANOVA with all pairwise multiple comparison procedures (Holm-Sidak method) (C and D), ~*p*<0.1, *p<0.05, **p<0.01, ***p<0.001. (E) Transmission electron microscope (TEM) analysis of TA muscles. The arrows are pointing at degenerative mitochondria. The cropped image is shown with 2.5x magnification of the original image. M: mitochondrion. Scale bars are 2 μm for the two first panels and 6 μm for the last.

Changes in locomotor capacity, muscle strength, and deregulation of the transcriptional control of energy metabolism led us to explore the muscle properties in Ts3Yah and Ms3Yah animals. We found an increased muscle mass in Ts3Yah animals and decreased muscle mass in Ms3Yah animals (Fig. 5A) that could account for the change in muscle strength observed in the animals. Gastrocnemius muscles are composed predominantly of type II glycolytic fibres and are poor in oxidative fast-twitch fibres that contain high amounts of mitochondria. We estimated the mitochondrial content of the gastrocnemius muscle by quantifying mtDNA and comparing the copy number of mtCOX2 normalised by nuclear DNA in transgenic animals versus their diploid control littermates. We observed a significant increase in mtDNA content in Ms3Yah muscles and close to significant decrease in Ts3Yah muscles (Student’s t-test Ts3Yah vs diploid, *p* = 0.06; Fig. 5B).

Mitochondrial activity was assessed by staining histological muscles sections for the presence of the mitochondrial enzyme succinate dehydrogenase (SDH). In tibialis anterioris (TA), a skeletal muscle rich in glycolytic fibres, the SDH-stained fibres versus nonstained fibres were counted. Ms3Yah muscles revealed significant increase in SDH-stained fibres compared to diploid and Ts3Yah muscles (Fig. 5C, top), but no change in the percentage of slow-twitch oxidative type I myofibers using MyHCI staining was observed (Fig. 5D, top). In the soleus, mainly composed of oxidative fibres, differences between highly oxidative and less oxidative fibres were determined by scoring darkly SDH-stained fibres from lightly stained fibres. A significant increase in the number of oxidative fibres was found in Ms3Yah compared to diploid and Ts3Yah muscles (Fig. 5C, bottom). An increased proportion of type I fibres was found in Ms3Yah compared to both diploid and Ts3Yah muscles. A decreased proportion of type I myofibers was also found in Ts3Yah soleus compared to diploid (Fig. 5D, bottom). Hence, we found that monosomy increased the oxidative state of the muscle while trisomy decreased it. We analysed the TA in electron microscopy. In skeletal muscle fibres, mitochondria are usually localised between myofibrils, aligned parallel to the myofibrils, and in pairs at the Z-disc of the sarcomere and packed strands underneath the sarcolemma. In Ts3Yah TA, some fibres (estimated <10%) were found having few small-sized mitochondria compared to normal fibres (Fig. 5E). In contrast, we could find areas with increased mitochondrial mass present between adjacent fibres and containing few degenerative mitochondria in Ms3Yah muscles (Fig. 5E). Overall, these results indicate increased muscle oxidative capacity in Ms3Yah muscles and decreased oxidative capacity in Ts3Yah muscles due to change in mitochondrial content.

### Analysis of mitochondrial function in skeletal muscles

**Fig 6:**
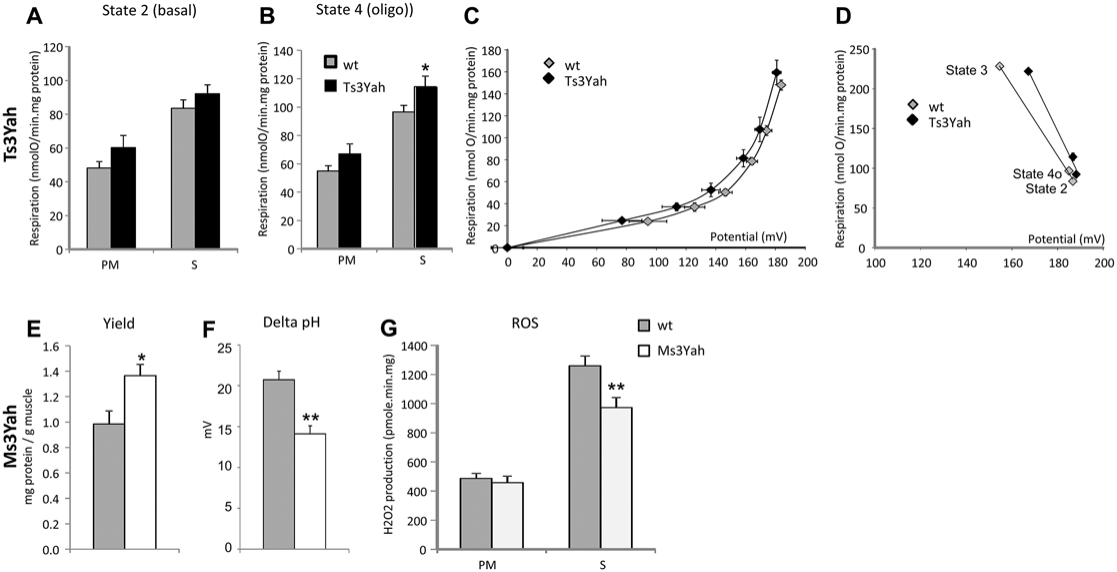
Evaluation of mitochondrial function. Mitochondria were isolated from hind limb muscle to test their respiration capacities. (A) Yield of mitochondrial protein per gram of muscle obtained from the mitochondrial extraction. (B–D) The rates of oxygen consumptions were measured in isolated muscle mitochondria in the presence of pyruvate/malate (PM) or succinate (S) as described in Materials and Methods. (B) State 2, basal was measured in the presence of substrates only. (C) State 3, ADP-stimulated respiration was measured with the addition of 500 μM ADP. (D) State 4o, after addition of oligomycin. (E) COX activity was given by measuring mitochondrial oxygen consumption during PM respiration with the addition of 10 mM antimycin, 2 mM ascorbate, and 0.5 mM TMPD. (F) Basal proton leak was visualised by plotting the rate of oxygen consumed against mitochondrial membrane potential. (G) Kinetic response of substrate oxidation activity to membrane potential at states 3, 2, and 4o in Ts3Yah and diploid isolated mitochondria. Data are presented as mean ± sem (n = 5–6 per genotype) with Student’s t-test (A and G) and two-way ANOVA with all pairwise multiple comparison procedures (Holm-Sidak method) (D), *p<0.05, **p<0.01.

To determine if mitochondrial function is altered in the skeletal muscles of Ts3Yah and Ms3Yah mice, we assessed mitochondrial functional capacity in isolated equal amounts of mitochondrial preparations from hind limb muscles. We measured mitochondrial respiration using pyruvate/malate (PM), whose breakdown products generate NADH and lead to the reduction of mitochondrial complex I and the citric acid intermediate succinate (S), which donates electrons to FAD-containing complex II of the electron transport chain. While Ts3Yah mitochondrial respiration rates during state 3 respiration with ADP was comparable to the respiratory rates of diploid mitochondria (S2A Fig), they were significantly higher compared to controls in both states 2 (basal respiration in the presence of substrate alone) (ANOVA ‘genotype,’ ‘substrate’; F[1, 13] = 3.674, *p* = 0.078) (Fig. 6A) and 4o (respiration in presence of oligomycin, an inhibitor of ATPase) (ANOVA ‘genotype,’ ‘substrate’; F[1, 13] = 56.681, *p* = 0.026) (Fig. 6B). The fact that the respiratory flux is increased in both states 2 and 4o in Ts3Yah mitochondria suggests an increase in the membrane permeability. The respiratory control ratio, defined as the respiration in state 3 (maximal ADP-stimulated respiration) divided by that in state 4o, remained unchanged, showing intact mitochondria (S2B Fig). The activity of cytochrome oxidase (COX), the terminal enzyme in the mitochondrial electron transport chain, was investigated by isolating its respiratory activity using antimycin and was not changed in Ts3Yah mitochondria (S2C Fig). To further explore proton leak in Ts3Yah mitochondria, we used the batch of mitochondria subjected to respiration with succinate and measured the Δψ_m_ in parallel with respiration in the absence of net ATP synthesis (presence of oligomycin) to calculate the basal proton conductance of the membrane [42]. A voltage plot for the conductance over a range of potentials was generated by starting in state 4o and progressively limiting electron transport by titrating malonate and simultaneously determining respiration and Δψ_m_. The curve of the relationship between oxygen consumption and membrane potential in Ts3Yah mitochondria was shifted to the left compared to diploid controls indicating that the rate of oxygen consumed to counteract proton leak was slightly increased (Fig. 6C). In addition, reporting the kinetic response of substrate oxidation activity to mitochondrial potential during states 2, 3, and 4o revealed increased respiratory activity in Ts3Yah compared to diploid controls as seen by the shift of the slope to the right (Fig. 6D). Finally, in non-phosphorylating conditions (state 4o), the mitochondrial potential in Ts3Yah mitochondria (186.7±1.9 mV) was the same as in diploid controls (185.0±2.2 mV), indicating that the higher rate of oxygen consumption observed in Ts3Yah was not due to an increase in oxidation of succinate. Together, those results suggest a slight change in the inner membrane proton conductance in Ts3Yah mitochondria. The mitochondrial respiration and COX activity in Ms3Yah mitochondria were similar to that of diploid controls (S2D–S2G Fig). However, the mitochondrial protein yield calculated from the reported amount of mitochondria extracted to the weight of muscle used for the extraction was significantly increased (37%; Student’s t-test, *p* = 0.024; Fig. 6E), suggesting that the increased oxidative capacity observed in Ms3Yah muscle is due solely to increased mitochondrial proliferation and not to increased mitochondrial respiration.

The kinetic analysis between oxygen consumption and membrane potential in Ms3Yah mitochondria was similar to that of diploid controls indicating a similar basal proton leak, as expected by the absolute respiratory rates observed in Ms3Yah mitochondria. However, although the basal proton motive force values were not significantly different between genotypes (Ms3Yah: 182 ± 3 mV; diploid control: 191±4 mV), the chemical component of the proton motive force ΔpH was significantly lower (~30%; t-test, *p* = 0.001) in Ms3Yah mice (14±1 mV) than in diploid controls (21±1 mV) (Fig. 6F). We also observed a 26% decrease in Ms3Yah mitochondrial ROS production (Fig. 6G, decrease in H_2_O_2_ release as a marker of ROS production) under basal respiration with S, which corresponds to the respiratory state that produces most ROS (ANOVA ‘genotype,’ ‘substrate’; F[1,31] = 6.068, *p* = 0.02, and post hoc tests with all pairwise multiple comparison procedure using Holm-Sidak method: Ms3Yah vs diploid with succinate as substrate *p* = 0.001). ROS production from S comes from a reverse electron flux from complex II to complex I (NAD) that produces superoxide at a very high rate and is highly dependent on the pH gradient (ΔpH) across the mitochondrial inner membrane [43]. Overall, our analysis revealed that there is a propensity of increased membrane permeability in Ts3Yah mitochondria and of decreased ROS production by Ms3Yah mitochondria that is due to a decrease of pH across the mitochondrial inner membrane.

## Discussion

The phenotypic characterisation of two new mouse models trisomic and monosomic for the 9.4 Mb Mmu16 *Hspa13*-*App* region syntenic to the centromeric 21q11.2-q11.3 region revealed the contribution of gene dosage to locomotion, muscle strength, and mass and energy balance. The locomotor deficits observed in DS were attributed to impaired coordination input due to cerebellar or more general central dysfunction. Structural anomalies have been observed in the cerebellum of DS patients [44–46], but the presence of other motor-related phenotypes such as increased time for feedback adjustments in grip force, higher static grip forces, and difficulties to maintain constant muscle power suggests a more complex origin [47, 48]. Studies on motor control have described specific preferential muscle coactivation patterns in people who have DS during voluntary movements or postural adjustments. This coactivation of the agonist-antagonist muscle pair might produce less resistance to motion and might contribute to hypotonia [49, 50].

The locomotor deficit in Ts3Yah is accompanied by increased grip strength but decreased muscle endurance. Functional characterisation of the soleus muscle of Ts65Dn mice revealed that the muscle weakness observed in vivo was not the result of a deficit in force-generating capacity of the muscle in the basal state but was rather due to post-fatigue muscle weakness [51]. Hence, hypotonia and motor deficits observed in DS might be associated more with the fatigability of the muscle due to a lower energetic state. Indeed, hypotonia corresponds to a state of low muscular tonicity as the consequence of lack of energy to keep working at a normal level for a long time (and of a slower speed of response [52, 53]. Lower exercise endurance capacity with diminished basal metabolism and aerobic capacity has been observed in DS persons [54–56]. Accordingly, walking anomalies observed in DS children are associated with difficulties to maintain constant muscle power [57]. Interestingly, our Ts3Yah mouse model shows locomotor deficits as a consequence of energy decrease and lack of endurance.

The contribution of skeletal muscles to DS locomotor phenotypes and their physiological and biochemical characterisation have been poorly explored. A recent analysis of the soleus muscle in Ts65Dn mice failed to highlight a clear change in mitochondrial content or in muscle fibre type composition in the muscle although it identified alterations in pathways involved in glucose and fat metabolism as well as ATP biosynthesis [51]. The examination of hormonal and metabolic responses after a maximal effort in a treadmill test in DS and age-matched control people revealed that exercise limitation in DS people was caused by limited aerobic capacity [56]. This limitation was attributed to the disruption of the hormonal response (cortisol and catecholamines) leading to an incapacity to control glycolysis, neoglucogenesis, and lipolysis when muscle exercise is pursued [56]. Altered mitochondrial activity and increased oxidative stress would play a similar role in muscular function as found in the central nervous system, with DS accelerated aging and neurodegeneration [58–61].

Ms3Yah mitochondria revealed a decreased ROS production under respiration with succinate that could be linked to a lower pH gradient (ΔpH) of the mitochondrial inner membrane, whereas no change was observed for the membrane electrical potential (*Δψ*). ROS production with succinate is mainly generated by reverse electron flux from complex II to complex I [62–65] and is at least threefold more sensitive to the pH gradient (ΔpH) across the mitochondrial inner membrane than to the membrane potential (*Δψ*) [43]. PH gradient can be generated by proton pumping during substrate oxidation or by the ATP synthase pumping protons into the mitochondrial matrix during ATP synthesis. Interestingly, *Atp5J*, which encodes the F6 subunit of the mitochondrial ATP synthase [66], is found in the *Hspa13-App* region and shows opposite deregulation in Ts3Yah and Ms3Yah mice. Change in the dosage of this subunit might lead to a change in ATP synthase efficiency and thus to a change in pH gradient across the mitochondrial inner membrane. Hence, transgenic mice overexpressing *Atp5J* have sustained a decrease in intracellular pH in tissues leading to acidosis [67]. A deficit of mitochondrial ATP production due to alteration of the catalytic OXPHOS capacity and associated to a compensatory enhancement of glycolysis and mitochondrial mass was seen in fibroblasts from DS subjects [68]. Increased respiration during non-phosphorylating conditions, an indicator of nonoptimal OXPHOS efficiency, was also observed in the same study and suggests a greater proton leak across the mitochondrial inner membrane of DS fibroblasts. Interestingly, as seen by Valenti and collaborators [68], we did not observe major changes in mitochondrial respiratory capacity in Ts3Yah and Ms3Yah mitochondria, but Ts3Yah mitochondria showed a slight increase in respiration during non-phosphorylating conditions and a shift in the relationship between oxygen consumption and mitochondrial membrane potential indicative of a greater proton leak. This finding, together with the presence in the *Hspa13-App* region of the gene coding for a subunit of the ATP synthase, suggest that ATP synthesis might be affected in Ts3Yah mitochondria. It would be interesting to further measure the ATPase activity as well as the ratio of ATP synthesis over oxygen consumption in Ts3Yah mitochondria.

The transcriptome analysis of Ts3Yah and Ms3Yah skeletal muscles identifies the deregulation of genes involved in energy metabolic pathways. These results are consistent with previous findings in the hearts of DS fetuses and in DS amniocytes [69, 70]. Moreover, our analysis suggests that *Pgc-1α* or members of the *Pgc-1α* pathway are important to the molecular changes observed in Ts3Yah and Ms3Yah muscles. Consistent with this hypothesis, we increased expression of *Pgc-1α* in the transcriptome of Ms3Yah muscles (FC = 1.25, *p* = 0.0027) and increased expression in Ms3Yah (FC = 1.18, *p* = 0.03) and decrease expression in Ts3Yah (FC = 0.81, *p* = 0.01) muscles of *ERRα* (ESRRA), encoding the orphan nuclear receptor ERRα (NR3B1) that is known to have co-expression with *Pgc-1α* in tissues with high energy demand and whose transcriptional activity has been extensively linked to members of the PGC-1 family of coactivators [71]. *Pgc-1α* is a transcriptional coactivator of steroid and nuclear receptors and was found to play a central role as a modulator of oxidative metabolism in response to external physiological stimuli by activating the expression of a wide array of genes involved in fuel intake, gluconeogenesis, and fatty acid oxidation [72]. Most importantly, *Pgc-1α* controls mitochondrial biogenesis through the regulation of the synthesis of mitochondrial proteins involved not only in oxidative phosphorylation [73, 74] but also in the mitochondrial ribosomal machinery, protein transport, and dynamic [75]. Interestingly, the gene encoding the mitochondrial enzyme citrate synthase (Cs) needed for pyruvate-derived acetyl-CoA entry to the TCA cycle and whose activity is a marker of increased mitochondrial biogenesis is found in the leading edge genes of the mitochondrial gene set identified in the GSEA analysis. In addition, genes coding for proteins involved in the transcription and translation of mitochondrial genes (*Tfam*, *Tufm*, *Tfb1m*, *Tfb2m*, *Lars2*, *Wars2*, *Mtif2*, and *Mtrf1*) and for proteins participating in mitochondrial protein import and translocation across the outer and inner mitochondrial membrane (*Mipep*,*Surf1*, *Tomm70a*, *Timm17b*, *Timm9*, *Timm44*, *and Tomm22*) were found in mitochondrial leading edge genes in either Ts3Yah or Ms3Yah or both (see leading edge gene set ‘HUMAN_MITODB_6_2002,’ S4–S5 Tables). Hence, molecular changes observed in Ts3Yah and Ms3Yah muscles point at gene(s) within the *Hspa13-App* region affecting the *Pgc1a* coactivator network regulating energy metabolism and mitochondrial biogenesis.

Eight of the 15 genes conserved between human and mouse in the *Hspa13-App* region, namely, *Hspa13*, *Nrip1*, *Usp25*, *Btg3*, *D16Ertd472e*, *Mrpl39*, *Jam2*, *Atp5J*, and *Gabpa*, were globally 0.5 fold downregulated in Ms3Yah muscles and 1.5 fold upregulated in Ts3Yah muscles following gene dosage. Among those genes, two (*Gabpa* and *Nrip1*) are strong candidates for the muscular phenotypes as they are known to be part of the network of proteins involved in the transcriptional control of cellular energy homeostasis. *Gabpa* (GA-binding protein subunit α) encodes the ETS DNA-binding subunit of the NRF2 (nuclear respiratory factor) transcription factor that was found to interact with *PGC-1α* and ERRα to drive the expression of mitochondrial genes [76, 77]. Genetic disruption and knockdown of mouse *Gabpa* caused early embryonic lethality [78, 79] with mitochondrial dysfunction and have been proposed as a possible explanation for embryonic loss. Yang and collaborators (2014) recently deleted *Gabpa* in cultured primary mouse fibroblasts and found out that this resulted in a reduction of mitochondrial mass, ATP production, and mitochondrial methyltransferase Tfb1m, which is essential for mitochondrial protein translation and mitochondrial biogenesis [80]. However, the Ms3Yah mice with only one copy of *Gabpa* have increased mitochondrial biogenesis, and the *Gabpα* heterozygous knockout mice display no phenotype and express similar levels of GABPα protein as the wild-type mice in their muscles [79]. Moreover, no increase in protein expression of GABPα could be seen in human DS fibroblasts [81].

The receptor-interacting protein (RIP140 or NRIP1) identified as a corepressor of nuclear receptors [82–84] was found to affect oxidative metabolism and mitochondrial biogenesis by negatively controlling mitochondrial pathways regulated by PGC-1α [85]. *Nrip1* null mice displayed an increase in the number of oxidative fibres as measured by SDH and MyHC isoforms staining and an increase in the quantity of mitochondria, whereas overexpression of NRIP1 reduced oxidative activity and mitochondrial biogenesis in skeletal muscle [86]. In addition, the absence of NRIP1 resulted in the upregulation of the same gene sets (oxidative phosphorylation, fatty acid oxidation, TCA cycle, and glycolysis) that we found deregulated in our models [86]. A siRNA experiment to decrease NRIP1 expression in trisomic human fetal fibroblasts enabled to recover normal levels of nuclear-encoded mitochondrial genes and normal mitochondrial function in those fibroblasts [87]. As such *Nrip1* changes in copy number might explain most of the phenotypes observed in Ts3Yah and Ms3Yah mice, but we cannot exclude the contribution of other genes from the interval. Indeed, the change in the steady-state level of a subunit of mitochondrial ribosomal protein (MRP) encoded by the *Mrpl39* gene within the *Hspa13-App* region may also trigger perturbation in the translation of mitochondrial-encoded proteins of the OXPHOS system, leading to variations of mitochondrial function observed in Ts3Yah and Ms3Yah mice. Translational deficiencies due to mutations in genes encoding MRPs have been described for four of the more than 70 MRPs, leading to growth retardation, cardiomyopathy, hypotonia, and brain anomalies [88–91] but are expected to range from lethality to slightly impaired energy metabolic deficiencies and give rise to various tissue-specific defects such as neuromuscular disorders or metabolic acidosis [92, 93]. In addition, deficit in the OXPHOS machinery can trigger the mitochondrial membrane permeability transition (MPT) leading to mitochondrial apoptosis or necrosis [94] as observed in Ms3Yah muscles.

DS and M21 are complex genetic diseases, and mouse models represent invaluable tools to decipher Hsa21 dosage-sensitive genes and associated deregulated molecular pathways. Our new models show that subtle deregulations contribute to the complexity of the DS-related pathology and indicate the need to further study the contribution of different Hsa21 regions to the modulation of DS. In particular, this study contributes to understanding the motor deficits in people who have DS by providing new insight into the effect of trisomy on muscle dysfunction and proposing new deregulated pathways that might represent novel therapeutic targets for DS therapy.

## Materials and Methods

### Ethics statement

All animals were treated in compliance with animal welfare policies from the French Ministry of Agriculture (Law 87 848 and YH accreditation 45–31 and 67–369). Mice were bred on C57BL/6J background for at least five generations prior to the functional analysis. The animals were bred under SPF conditions and were treated in compliance with animal welfare policies. In all the experiments, disomic littermates matched for age and gender were used as controls. A series of behavioural experiments were conducted in order to evaluate motor and muscular functions. All experimental procedures have been described previously in [36]. Behavioural protocols (rotarod, grip strength, treadmill, notched bar, and open field) were approved by the local Ethics Committee for Animal Experimentation under the accreditation number (2012–069). For all these tests, mice were kept in SPF conditions with free access to food and water. The light-dark cycle was 12:12, with lights on at 7:00 a.m. All the tests were done between 9:00 a.m. and 4:00 p.m.

After weaning, male mice were gathered by litters in the same cage. The different apparatus used were placed in a dimly lit testing room (approximatively 60 lux). To produce experimental groups, only animals coming from litters containing a minimum of two male pups were selected. Groups of animals were transferred to the experimental room 30 minutes before each experimental test. No invasive procedure was used, and the mice were euthanized at the end of the analysis.

### Generating the Ts3Yah and Ms3Yah mouse strains

The Ms3Yah monosomic mice, Del(16*Stch*-*App*)3Yah, and the Ts3Yah trisomic mice, Dp(16*Stch*-*App*)2Yah, were generated by chromosomal engineering in vivo as described in more details in S1 Text. Briefly, *Hspa13* and *App* targeting vectors containing loxP sites and isolated from *5’Hprt* and *3’Hprt* libraries [95] were inserted by homologous recombination in HM1–1 *Hprt*-deficient ES cells in a trans configuration [96] (Fig. 1B–C). Transient expression of the Cre recombinase generated clones with the tandem duplication and the deletion of the *Hspa13-App* fragment (Fig. 1D) that were subsequently injected to C57BL/6J blastocysts to generate chimeras. Chimeras were mated with C57Bl6/J animals and pups carrying either the Mmu16 with the deletion or the Mmu16 with the duplication that were identified by Southern blot analysis. Those animals were subsequently bred with C57BL/6J mice to obtain the Ms3Yah and Ts3Yah lines.

### Behavioural analyses

Groups (n = 10–15) of transgenic and diploid littermate males with matched age and genetic background (B6 from N5 backcross level) were used for the different behavioural tests, following the recommendations from the standard operating procedures developed by the Eumorphia network (http://www.eumorphia.org). A series of behavioural experiments were conducted in order to evaluate the motor conditions in mice with group of 10 to 15 males per genotype. For all these tests, mice were kept in SPF conditions with free access to food and water. Several tests were carried out, including open-field, rotarod, notched bar,and grip strength at ages 3 to 4 months and rotarod, grip strength, treadmill endurance, and notched bar at ages 8 to 12 months. All procedures, except for the treadmill exercise, are presented in S1 text. The treadmill system consists of a belt, which is enclosed in a Plexiglas chamber, and a stimulus device with a metal shock grid attached to the rear of the belt. The speed and slope of the belt are electronically adjusted. The animals were acclimatised with a 20-minute run at 25 cm/s and a 5-degree incline the day before the running test. For the actual test, the experiment was started at 25 cm/s and a 5-degree incline. Every 20 minutes, a progressive increase of 5 cm/s in speed is applied. The distance run and the time spent running were recorded, and a mouse was considered exhausted and removed from the experiment if it received approximately 100 shocks (1 mA) in a period of 5 minutes or if it was spending more than 20 seconds on the shock grid. The duration of running and the total distance covered evaluate the performance of the mice.

### Gene expression analysis

#### RNA extraction

Total RNA was extracted from frozen gastrocnemius muscles from Ms3Yah and Ts3Yah and their respective control diploid littermates (n = 6) using TRIzol Reagent (Invitrogen) followed by RNase-Free DNaseI (Qiagen) treatment.

#### Microarray hybridisation and data analysis

Biotinylated cDNA were prepared from total RNAs and hybridised to Illumina’s MouseWG-6 v2.0 Expression BeadChips. Raw data was normalised by the quantile method [97], and values were log transformed. A principal component analysis was done using the GeneSpring software (Agilent Technologies) and showed that one diploid sample corresponding to the Ts3Yah control group had a completely different profile. This sample was excluded from the analysis. The data were analysed separately for Ms3Yah and Ts3Yah, comparing each set to its own littermate diploid group. The genes expressed above the background level were determined by selecting the probe sets having a signal value above the 35th percentile of all expression values in at least one array. This corresponded to a threshold value above 90 in raw data (6.49 in log scale). The genes differentially expressed were selected by statistical analysis (Student’s t-test *p*<0.05 between transgenic mice and their respective diploid littermates). Verification was done to ensure that selected probes have acceptable false discovery rate (<10%, [98]), and resampling analysis was used to make 1,000 permutations of the samples for each probe in order to calculate the false discovery rate *p*-value (Zoe software; http://www-microarrays.u-strasbg.fr/base.php?page=analysisExpressionFilterZoeE.php). FC was calculated as a ratio of averages from transgenic and diploid signals with values <1 representing probes that are underexpressed in the transgenic (Ts3Yah or Ms3Yah) compared to diploid controls and values >1 representing probes that are overexpressed in the transgenic compared to the diploid controls.

Hierarchical clustering was carried out on significantly deregulated genes (*p*<0.05) with Cluster 3.0 software [99] using Euclidian distances to calculate the distances between the genes and between the samples. The calculated distances were then clustered by complete linkage clustering. The red-to-green colour scale represents the mean-adjusted expression values, where red corresponds to higher expression and green to lower expression.

Gene Set Enrichment Analysis (GSEA) was used to test sets of related genes that might be coordinately deregulated in Ts3Yah and Ms3Yah gastrocnemius muscles [40, 41]. The whole transcriptome output lists of Ts3Yah and Ms3Yah differentially expressed genes, together with their diploid controls, were submitted to the GSEA tool, and the functional gene sets containing curated biological pathways from the MSigDB C2 curated database were tested [40]. A number of 1,000 permutations were specified as recommended in order to assess the statistical significance of the enrichment score (ES). The GSEA analysis report highlights enrichment gene sets with an FDR<5%. All data are accessible under the GEO accession number (GSE58463) [NCBI tracking system #17057227].

### QRT-PCR

cDNA synthesis was performed using the SuperScript III First-Strand Synthesis SuperMix for QRT-PCR (Invitrogen). A series of primer pairs (available upon request) were designed to span intron-exon junctions. Efficiencies of the TaqMan assays were checked using a cDNA dilution series from the extracts of gastrocnemius muscle samples. The QPCR was performed with 300 nM of each primer and 100 nM of HPLC purified FAM-TAMRA-labelled double-dye TaqMan probes in a final reaction of 15 μl with a standard amplification procedure. Normalisation was performed by carrying out in parallel the amplification of five housekeeping genes (*Actb*, *Pgk1*, *Hprt1*, *Ppia*, and *Gnas*) and by using the GeNorm procedure [100] in order to correct the variations of the amount of source RNA in the starting material. All the tested samples were performed in triplicate, and the results were reported as the mean ± sem.

### mtDNA quantification by quantitative real-time PCR

DNA was isolated from muscle tissue, and quantitative PCR using the TaqMan technology was performed in triplicate to determine the relative quantity of the mitochondrial DNA marker cyclooxygenase 2 (Cox2) and the genomic DNA marker myxovirus resistance 1 (Mx1) gene. mtCOX2 and Mx1 primers were purchased from Sigma-Aldrich and TaqMan MGB probes for mtCOX2 and Mx1 from Applied Biosystem. PCR conditions were as follows: (1) 50°C for 2 minutes, (2) 95°C for 10 minutes, (3) 95°C for 15 seconds, and (4) 60°C for 1 minute (steps 3 and 4 were repeated 50 times). The results were calculated from the difference in threshold cycle (ΔCT) values for mtDNA and nuclear-specific amplification. Data were expressed as mtDNA in transgenic mice relative to diploid control groups.

### SDH and immunofluorescence staining on frozen muscle sections

Tibialis anterioris muscles and soleus tissues were collected from 5 to 7 mice aged 4 to 6 months per genotype and immediately frozen in 5 minutes and 3 minutes, respectively, in isopentane cooled in liquid nitrogen (-190°C). A total of 10 μm thick serial sections were obtained using a cryostat (Leica CM3050) at -25°C. The sections were processed for succinate dehydrogenase (SDH) staining (S1 Text). Alternatively, type I fibres in tibialis and soleus were characterised by MyHC-I immunostaining using the primary monoclonal mouse antibody against type I (M844, Sigma) as described in S1 Text. Quantification was done by counting muscle fibres using the ImageJ software (W. Rasband, NIH; http://rsb.info.nih.gov/ij/) on at least three consecutive sections.

### Electron microscopy

TA samples (n = 3–5) of 3-month and 12-month old mice were fixed in 2.5% glutaraldehyde and 2.5% paraformaldehyde in cacodylate buffer (0.1 M, pH 7.4), postfixed in 1% osmium tetroxide in 0.1M cacodylate buffer, and dehydrated through graded alcohol (50%, 70%, 90%, and 100%) and propylene oxide. The samples were oriented longitudinally and embedded in Epon 812. Semithin sections were cut at 2 μm, and ultrathin sections were cut at 70 nm and contrasted with uranyl acetate and lead citrate and examined at 70 kv with a Morgagni 268D electron microscope. The images were captured digitally by a MegaView III camera (Soft Imaging System).

### Analysis of mitochondrial function

Mitochondria were isolated from hind limb skeletal muscles from one or two adult mice per mitochondrial preparation as described in S1 Text. The protein concentration of mitochondrial suspensions was determined in duplicate by a biuret method with bovine serum albumin to normalise the samples. Oxygen consumption was measured with a Clark oxygen electrode (Rank Brothers Ltd) in a stirred and closed chamber as described in S1 text. The cytochrome-c oxidase (COX) activity was determined by the mitochondrial respiration rate in the mitochondrial suspension, which includes 10 mM antimycin, 2 mM ascorbate, and 0.5 mM TMPD (N,N,N’,N’-TetraMethyl-p-Phenylene-Diamine). Measurements of COX activity were done in duplicate on n = 5 per genotype.

Mitochondrial membrane potential was measured using an electrode sensitive to the lipophilic cation triphenylmethylphosphonium (TPMP^+^). The protocol described in S1 Text allows us to calculate the ΔpH value as the difference between membrane potential values in the presence (ΔpH + Δψ) and in the absence (Δψ) of nigericin.

Respiration and membrane potential were then progressively inhibited through successive steady states induced by the addition of malonate. After each run, 2 μM FCCP was added to release TPMP^+^ back into the medium for baseline correction. Membrane potentials were calculated as described previously [101], assuming a TPMP^+^ binding correction of 0.35 mg protein/μl for skeletal muscle mitochondria [102].

Radical oxygen species (ROS) production by isolated mitochondria was evaluated by measuring the rate of H_2_O_2_ released using an SFM-25 fluorometer (Kontron) at excitation and emission wavelengths of 560 nm and 584 nm, respectively. The respiratory buffer (1 ml) was supplemented with 5 U/ml horseradish peroxidase, 1 μM Amplex Red reagent, and mitochondria (15–20 μg/ml). Respiratory substrates (5 mM succinate or 5 mM pyruvate/2.5 malate or 40 μM palmitoyl-L-carnitine/2.5 malate) were added to start the reaction. The fluorescent signal was calibrated using a standard curve obtained after successive addition of H_2_O_2_ (20 to 80 pmoles).

### Statistics

Unless otherwise stated, statistical analyses were performed comparing two groups, the Ts3Yah or Ms3Yah and their diploid control littermates, using Student’s t-test when appropriate or the nonparametric Mann-Whitney rank sum test. For tests requiring serial recordings, data were analysed by two-way ANOVA using the SigmaPlot software. Data are presented as mean ± sem. Significant threshold was set for *p*<0.05.

### Supplemental information

Supplemental information includes additional experimental procedures, two figures, and five tables and can be found online.

## Supporting Information

S1 Table Orthologous genes on Mmu16 and Hsa21 *Hspa13-App* region. (DOCX)

S2 Table Ts3Yah vs diploid littermates. Genes whose expression is significantly altered in Ts3Yah gastrocnemius muscles with FC>|1.2| (t-test *p*<0.05). Gene names from the *Hspa13-App* region are in italics. Probes deregulated in Ms3Yah model are in bold.
(DOCX)

S3 Table Ms3Yah vs diploid littermates. Genes whose expression is significantly altered in Ms3Yah gastrocnemius muscles with FC>|1.2| (t-test *p*<0.05). Gene names from the *Hspa13-App* region are in italics. Probes deregulated in Ms3Yah muscles are in bold.
(DOCX)

S4 Table Gene set enrichment analysis in diploid vs Ts3Yah. List of significantly enriched gene sets as indicated by false discovery rate (FDR) <5%. Genes sets with negative enrichment score (gene sets that show enrichment at the bottom of the ranked list, corresponding to downregulated genes compared to diploid controls) are listed for the Ts3Yah line as there was no gene set with FDR<5% in the list of gene sets with positive enrichment score. Set size: number of genes in the gene set after filtering out genes not in the expression data set. ES: enrichment score for the gene set, representing the degree to which the gene set is overrepresented at the edges (top or bottom) of the ranked list in the expression dataset. NES: normalised enrichment score, ES after normalisation across analysed gene sets. Nom *p*-value: nominal *p* value giving the statistical significance of the enrichment score. FDR *q*-value: false discovery rate giving the probability that NES represent a false positive.
(DOCX)

S5 Table Gene set enrichment analysis in Ms3Yah vs diploid. List of significantly enriched gene sets as indicated by false discovery rate (FDR) <5%. Gene sets with positive enrichment score (gene sets that show enrichment at the top of the ranked list, corresponding to upregulated genes compared to diploid controls) are given for the Ms3Yah line that had no gene set with a significant negative enrichment score. Set size: number of genes in the gene set after filtering out genes not in the expression data set. ES: enrichment score for the gene set representing the degree to which the gene set is overrepresented at the edges (top or bottom) of the ranked list in the expression dataset. NES: normalised enrichment score, ES after normalisation across analysed gene sets. Nom *p*-value: nominal *p* value giving the statistical significance of the enrichment score. FDR *q*-value: false discovery rate giving the probability that NES represent a false positive.
(DOCX)

S1 Fig Evaluation of motor skills. (A) Rotarod tests at incremental fixed speed (repeated ANOVA ‘genotype,’ ‘speed’: Ts3Yah vs diploid: F[1, 69] = 2.395 *p* = 0.135; Ms3Yah vs diploid: F[1, 90] = 2.607 *p* = 0.117) and at accelerating speed (two trials [4–40 rpm] in five minutes with the mean rotational velocity at the time of falling calculated for the two trials). (B) Endurance to exercise was measured as the maximal running distance travelled on a treadmill until exhaustion. (C) Spontaneous locomotor activity and habituation were assessed in an open field by measuring the distance travelled during the first 15 minutes and the last 15 minutes of a 30-minute session. (D) Motor coordination skills were tested with a notched bar by looking at the percentage of errors made by the mouse with its hind paws when crossing the notched bar. Data are presented as mean ± sem.
(TIF)

S2 Fig Evaluation of mitochondrial function. Mitochondria were isolated from hind limb muscle to test their respiration capacities. (A) State 3, ADP-stimulated respiration in Ts3Yah mitochondria. (B) Respiratory control ratio (RCR) is the ratio of state 3 to state 4o. (C) COX activity was monitored by measuring mitochondrial oxygen consumption during PM respiration with the addition of 10 mM antimycin, 2 mM ascorbate, and 0.5 mM TMPD. Yield of mitochondrial protein per gram of muscle is obtained from the mitochondrial extraction. (D–F) Rates of oxygen consumption in Ms3Yah mitochondria measured in the presence of pyruvate/malate (PM) or succinate (S). (D) State 2, basal was measured in the presence of substrates only. (E) State 3, ADP-stimulated respiration (ADP) was measured with the addition of 500 μM ADP. (F) State 4o, after addition of oligomycin. (G) COX activity in Ms3Yah mitochondria. Data are presented as mean ± sem (n = 5–6 per genotype).
(TIF)

S1 Text Supporting information about gene targeting and generation of chromosomal rearrangements in ES cells; Southern blot analysis; Fluorescent in situ hybridisation; Array-based comparative genomic hybridisation; Open field; Rotarod test; Notched bar test; Grip strength; SDH on muscle sections; Immunofluorescence staining (fibre typing); Isolation of skeletal muscle mitochondria; Mitochondrial respiration and COX activity; Mitochondrial membrane potential. (DOCX)
