# Supplementary material for: Opposite Phenotypes of Muscle Strength and Locomotor Function in Mouse Models of Partial Trisomy and Monosomy 21 for the Proximal Hspa13-App Region
Source: PLoS Genet. 2015 Mar 24;11(3):e1005062. doi: 10.1371/journal.pgen.1005062 (PMC4372517; doi:10.1371/journal.pgen.1005062)
Supplement: S1 Table — (DOCX) [file pgen.1005062.s001.docx]

| **Names of mouse genes** | **Genome coordinates (Start position)** | **Names of human genes** | **Genome coordinates (Start position)** |
| --- | --- | --- | --- |
| *Hspa13* | 75755190 | *HSPA13* | 143711115 |
| *Samsn1* | 75858793 | *SAMSN1* | 14485228 |
| *Nrip1* | 76287400 | *NRIP1* | 14961235 |
| *Usp25* | 77014069 | *USP25* | 15730025 |
| *Cxadr* | 78301496 | *CXADR* | 17512382 |
| *Btg3* | 78359860 | *BTG3* | 17593653 |
| *D16Ertd472e* | 78544012 | *C21orf91* | 17788967 |
| *Chodl* | 78930948 | *CHODL* | 17901263 |
| *Prss7 (Tmprss15)* | 78953008 | *PRSS7 (TMPRSS15)* | 18269116 |
| *Ncam2* | 81200697 | *NCAM2* | 20998315 |
| *Mrpl39* | 84717576 | *MRPL39* | 25585656 |
| *Jam2* | 84774123 | *JAM2* | 25639272 |
| *Atp5j* | 84827866 | *ATP5J* | 25716503 |
| *Gabpa* | 84834925 | *GABPA* | 25734570 |
| *App* | 84954440 | *APP* | 25880550 |
