# Supplementary material for: Opposite Phenotypes of Muscle Strength and Locomotor Function in Mouse Models of Partial Trisomy and Monosomy 21 for the Proximal Hspa13-App Region
Source: PLoS Genet. 2015 Mar 24;11(3):e1005062. doi: 10.1371/journal.pgen.1005062 (PMC4372517; doi:10.1371/journal.pgen.1005062)
Supplement: S2 Table — Genes whose expression is significantly altered in Ts3Yah gastrocnemius muscles with FC>|1.2| (t-test p<0.05). Gene names from the Hspa13-App region are in italics. Probes deregulated in Ms3Yah model are in bold. (DOCX) [file pgen.1005062.s002.docx]

| **Symbol** | **Probe ID** | **Gene name** | **Chromosome location** | **Fold change** | **P-value** |
| --- | --- | --- | --- | --- | --- |
| *9830131B04Rik (Ttn)* | ILMN_1223811 | Titin | 2 | 1.939 | 0.034 |
| *4930455C21Rik (Timmdc1)* | ILMN_2838965 | RIKEN cDNA 4930455C21 gene (translocase of inner mitochondrial membrane domain containing 1) | 16 | 1.832 | 0.027 |
| *Mtap4* | ILMN_2633367 | microtubule-associated protein 4 | 9 | 1.670 | 0.036 |
| *Gm26917* | ILMN_2638923 | predicted gene, 26924 | 17 | 1.657 | 0.039 |
| *gi_39893_emb_X17013.1_BSDPD_1251* | ILMN_2459100 |  |  | 1.600 | 0.046 |
| *Eif4ebp2* | ILMN_2673566 | eukaryotic translation initiation factor 4E binding protein 2 | 10 | 1.594 | 0.012 |
| *2900060B14Rik* | ILMN_1253544 | RIKEN cDNA 2900060B14 gene | 1 | 1.586 | 0.024 |
| *18S_rRNA_X00686_301* | ILMN_1230073 | 18S RNA |  | 1.581 | 0.031 |
| *Cacna2d1* | ILMN_2752030 | calcium channel, voltage-dependent, alpha2/delta subunit 1 | 5 | 1.563 | 0.048 |
| *4933421G18Rik* | ILMN_1234693 | Nipped-B homolog (Drosophila) |  | 1.529 | 0.039 |
| *Pbx1* | ILMN_2559669 | pre B-cell leukemia transcription factor 1 | 1 | 1.518 | 0.042 |
| *Pdap1* | ILMN_2654932 | PDGFA associated protein 1 | 5 | 1.512 | 0.021 |
| *Mef2c* | ILMN_1214950 | myocyte enhancer factor 2C | 13 | 1.494 | 0.006 |
| *2310050B05Rik* | ILMN_1244715 | RIKEN cDNA 2310050B05 gene | 10 | 1.492 | 0.048 |
| *2600001B17Rik (Lsm12)* | ILMN_2730926 | LSM12 homolog (S. cerevisiae) | 11 | 1.489 | 0.018 |
| *B430305P08Rik (CD47)* | ILMN_1246772 | CD47 antigen (Rh-related antigen, integrin-associated signal transducer) | 7 | 1.482 | 0.002 |
| *Copeb/Klf6* | ILMN_2731949 | Kruppel-like factor 6 | 13 | 1.459 | 0.034 |
| ***Chodl*** | ***ILMN_1254082*** | ***chondrolectin*** | ***16*** | ***1.458*** | ***0.002*** |
| *Pcyt1a* | ILMN_2688728 | phosphate cytidylyltransferase 1, choline, alpha isoform |  | 1.457 | 0.011 |
| *Ddx24* | ILMN_2747381 | DEAD (Asp-Glu-Ala-Asp) box polypeptide 24 | 12 | 1.454 | 0.030 |
| *Nfic* | ILMN_2710678 | nuclear factor I/C | 10 | 1.453 | 0.023 |
| *Tomm22* | ILMN_2507182 | predicted gene 12906; predicted gene 7250; translocase of outer mitochondrial membrane 22 homolog (yeast) | 15 | 1.450 | 0.030 |
| *Ear11* | ILMN_2890019 | eosinophil-associated, ribonuclease A family, member 11 | 14 | 1.445 | 0.045 |
| *Tcf25* | ILMN_3038404 | transcription factor 25 (basic helix-loop-helix) | 8 | 1.437 | 0.047 |
| *4833414E09Rik* | ILMN_2748880 |  | 1 | 1.419 | 0.017 |
| *Csnk2a1-rs3* | ILMN_1236941 | casein kinase 2, alpha 1 polypeptide; predicted gene 10031; similar to casein kinase II, alpha 1 polypeptide | 17 | 1.418 | 0.043 |
| *Mgl2* | ILMN_2707319 | macrophage galactose N-acetyl-galactosamine specific lectin 2 | 11 | 1.415 | 0.020 |
| *Sms* | ILMN_1217489 | predicted gene 7270; predicted gene 14680; spermine synthase | 3 | 1.411 | 0.044 |
| *Keap1* | ILMN_2621346 | kelch-like ECH-associated protein 1 | 9 | 1.399 | 0.00003 |
| *Lsm12* | ILMN_2963580 | LSM12 homolog (S. cerevisiae) | 11 | 1.398 | 0.0007 |
| ***Atp5j*** | ***ILMN_2611261*** | ***ATP synthase, H+ transporting, mitochondrial F0 complex, subunit F*** | ***16*** | ***1.396*** | ***0.00003*** |
| *3010027A04Rik (Ddx6)* | ILMN_2727235 | ankyrin repeat domain 11 / DEAD (Asp-Glu-Ala-Asp) box polypeptide 6 | 9 | 1.386 | 0.048 |
| *Keap1* | ILMN_2707771 | kelch-like ECH-associated protein 1 | 9 | 1.371 | 0.001 |
| *Rragd* | ILMN_2775202 | Ras-related GTP binding D | 4 | 1.3670 | 0.007 |
| *Map3k7ip2* | ILMN_2679094 | mitogen-activated protein kinase kinase kinase 7 interacting protein 2 | 10 | 1.369 | 0.005 |
| *Pctk1* | ILMN_2757150 | PCTAIRE-motif protein kinase 1 | X | 1.367 | 0.016 |
| *Ddx6* | ILMN_2606667 | DEAD (Asp-Glu-Ala-Asp) box polypeptide 6 | 9 | 1.366 | 0.039 |
| *Wnt4* | ILMN_2512663 | wingless-related MMTV integration site 4 | 4 | 1.361 | 0.001 |
| *Mef2c* | ILMN_2887992 | myocyte enhancer factor 2C | 13 | 1.358 | 0.006 |
| ***LOC654426 (Atp5j)*** | ***ILMN_2876629*** | ***ATP synthase, H+ transporting, mitochondrial F0 complex, subunit F pseudogene*** | ***16*** | ***1.340*** | ***0.0002*** |
| *Tm4sf3* | ILMN_1220261 | tetraspanin 8 | 10 | 1.332 | 0.045 |
| *F830009M01Rik (Rbbp4)* | ILMN_1249638 | retinoblastoma binding protein 4 | 4 | 1.330 | 0.034 |
| *2310040G07Rik* | ILMN_1232245 |  | 5 | 1.324 | 0.023 |
| *Eif2c2* | ILMN_2609056 | eukaryotic translation initiation factor 2C, 2 | 15 | 1.318 | 0.003 |
| *Wnt4* | ILMN_2889641 | wingless-related MMTV integration site 4 | 4 | 1.308 | 0.014 |
| *1300018P11Rik (Eif4e3)* | ILMN_2643291 | eukaryotic translation initiation factor 4E member 3 | 6 | 1.306 | 0.030 |
| ***Atp5j*** | ***ILMN_2632890*** | ***ATP synthase, H+ transporting, mitochondrial F0 complex, subunit F*** | ***16*** | ***1.305*** | ***0.0009*** |
| *Atp8a1* | ILMN_2509817 | ATPase, aminophospholipid transporter (APLT), class I, type 8A, member 1 | 5 | 1.302 | 0.044 |
| *Twistnb* | ILMN_2466121 | predicted gene 8670; TWIST neighbor | 12 | 1.301 | 0.031 |
| *Ank1* | ILMN_1236869 | ankyrin 1, erythroid; hypothetical protein LOC100046690 | 8 | 1.297 | 0.021 |
| *Mnab* | ILMN_1251986 | ring finger and CCCH-type zinc finger domains 2 | 2 | 1.297 | 0.020 |
| *1500031N17Rik (Lsm6)* | ILMN_1221336 | LSM6 homolog, U6 small nuclear RNA associated (S. cerevisiae) | 8 | 1.294 | 0.005 |
| ***Atp5j*** | ***ILMN_2962632*** | ***ATP synthase, H+ transporting, mitochondrial F0 complex, subunit F*** | ***16*** | ***1.291*** | ***0.0001*** |
| *B230387C07Rik* | ILMN_1216085 | ankyrin repeat domain 12; similar to Ankrd12 protein | 17 | 1.290 | 0.013 |
| *BC026370* | ILMN_1256171 | transmembrane protein 63b | 17 | 1.286 | 0.023 |
| *Eif2b5* | ILMN_2602140 | eukaryotic translation initiation factor 2B, subunit 5 epsilon | 16 | 1.285 | 0.001 |
| *Eif2b5* | ILMN_2930067 | eukaryotic translation initiation factor 2B, subunit 5 epsilon | 16 | 1.281 | 0.003 |
| *Gabarapl1* | ILMN_1236958 | gamma-aminobutyric acid (GABA) A receptor-associated protein-like 1 | 6 | 1.281 | 0.013 |
| *Rab1b* | ILMN_2693124 | RAB1B, member RAS oncogene family | 19 | 1.276 | 0.006 |
| *Stac3* | ILMN_2724841 | SH3 and cysteine rich domain 3 | 10 | 1.276 | 0.023 |
| *B230208H17Rik (Rabl6)* | ILMN_1223447 | RIKEN cDNA B230208H17 gene/RAB, member of RAS oncogene family-like 6 | 2 | 1.276 | 0.028 |
| *Nfic* | ILMN_3022492 | nuclear factor I/C | 10 | 1.275 | 0.002 |
| *Mpra* | ILMN_1222036 | progestin and adipoQ receptor family member VII | 4 | 1.275 | 0.008 |
| ***D16Ertd472e*** | ***ILMN_1260073*** | ***DNA segment, Chr 16, ERATO Doi 472, expressed*** | ***16*** | ***1.268*** | ***0.0002*** |
| *Ptp4a2* | ILMN_2725835 | predicted gene 13422; protein tyrosine phosphatase 4a2 | 4 | 1.268 | 0.023 |
| *2600005C20Rik (Rrp1)* | ILMN_2595283 | ribosomal RNA processing 1 homolog B (S. cerevisiae) | 17 | 1.264 | 0.012 |
| *Hsd3b2* | ILMN_2898958 | hydroxy-delta-5-steroid dehydrogenase, 3 beta- and steroid delta-isomerase 2 | 3 | 1.263 | 0.022 |
| *Mef2c* | ILMN_2732465 | myocyte enhancer factor 2C | 13 | 1.258 | 0.028 |
| *Pacsin3* | ILMN_2628066 | protein kinase C and casein kinase substrate in neurons 3 | 2 | 1.257 | 0.035 |
| *Kif1c* | ILMN_1226888 | kinesin family member 1C | 11 | 1.257 | 0.008 |
| *1700120C14Rik* | ILMN_1258006 | RIKEN cDNA 1700120C14 gene | 15 | 1.256 | 0.027 |
| *4921517L17Rik* | ILMN_2730831 | RIKEN cDNA 4921517L17 gene | 2 | 1.253 | 0.040 |
| *LOC385825* | ILMN_2537782 |  |  | 1.251 | 0.0001 |
| *Pfdn2* | ILMN_1219667 | prefoldin 2 | 1 | 1.249 | 0.048 |
| *Twistnb* | ILMN_1231779 | predicted gene 8670; TWIST neighbor | 12 | 1.248 | 0.021 |
| *Dag1* | ILMN_2853957 | dystroglycan 1 | 9 | 1.246 | 0.015 |
| *Prkaa2* | ILMN_1227113 | protein kinase, AMP-activated, alpha 2 catalytic subunit | 4 | 1.243 | 0.026 |
| *AI838661 (Gpn2)* | ILMN_1214375 | GPN-loop GTPase 2 | 4 | 1.242 | 0.001 |
| *5830445O15Rik (Ganc)* | ILMN_1226527 | glucosidase, alpha; neutral C | 2 | 1.241 | 0.005 |
| *1110063F24Rik (Bsdc1)* | ILMN_1234100 | BSD domain containing 1 | 4 | 1.240 | 0.015 |
| *Ptpre* | ILMN_1254630 | protein tyrosine phosphatase, receptor type, E | 7 | 1.237 | 0.034 |
| *B930008G03Rik* | ILMN_2565835 | predicted gene 10125 | 18 | 1.235 | 0.011 |
| *A730096C04Rik* | ILMN_2562439 | predicted gene 3014 | 8 | 1.234 | 0.047 |
| *Ncoa6ip (Tgs1)* | ILMN_2588456 | predicted gene 5117; trimethylguanosine synthase homolog (S. cerevisiae) | 4 | 1.233 | 0.009 |
| *2010007K12Rik* | ILMN_1220626 | KRIT1, ankyrin repeat containing | 5 | 1.230 | 0.016 |
| *Raf1* | ILMN_1237730 | v-raf-leukemia viral oncogene 1 | 6 | 1.228 | 0.049 |
| *2510048K03Rik (Prcp)* | ILMN_2639155 | prolylcarboxypeptidase (angiotensinase C) | 7 | 1.226 | 0.038 |
| *Cdk11b* | ILMN_1241980 | cyclin-dependent kinase 11B | 4 | 1.225 | 0.012 |
| *H13* | ILMN_2700292 | histocompatibility 13 | 2 | 1.224 | 0.033 |
| *Lyrm2* | ILMN_2739340 | LYR motif containing 2 | 4 | 1.223 | 0.002 |
| *LOC382157* | ILMN_2542231 |  | 5 | 1.218 | 0.035 |
| *F13a1* | ILMN_2914938 | coagulation factor XIII, A1 subunit | 13 | 1.217 | 0.019 |
| *Snrp70* | ILMN_2546888 | small nuclear ribonucleoprotein 70 (U1) | 7 | 1.215 | 0.029 |
| *Zfp91* | ILMN_3153893 | Zfp91-Cntf readthrough transcript; zinc finger protein 91; ciliary neurotrophic factor | 19 | 1.213 | 0.017 |
| *Hsd11b1* | ILMN_3115917 | hydroxysteroid 11-beta dehydrogenase 1 | 1 | 1.208 | 0.018 |
| *Odz3* | ILMN_2741231 | odd Oz/ten-m homolog 3 (Drosophila) | 8 | 1.208 | 0.012 |
| *Rnf10* | ILMN_2789888 | ring finger protein 10 | 5 | 1.207 | 0.0004 |
| *Farp2* | ILMN_2745925 | FERM, RhoGEF and pleckstrin domain protein 2 | 1 | 1.205 | 0.002 |
| *1810008O21Rik (Tmem160)* | ILMN_1219656 | transmembrane protein 160 | 7 | 1.205 | 0.004 |
| *9830123M21Rik (Igfn1)* | ILMN_1227447 | immunoglobulin-like and fibronectin type III domain containing 1 | 1 | 1.205 | 0.045 |
| *Usp37* | ILMN_1235652 | ubiquitin specific peptidase 37 | 1 | 1.203 | 0.023 |
| *BC017647* | ILMN_1233664 | cDNA sequence BC017647 | 11 | 1.203 | 0.015 |
| *H2afy* | ILMN_1247199 | H2A histone family, member Y | 13 | 1.201 | 0.004 |
| *2810410P22Rik (Arl5a)* | ILMN_1221102 | ADP-ribosylation factor-like 5A | 2 | 0.798 | 0.036 |
| *mtDNA_ND3* | ILMN_2495796 | mitochondrially encoded NADH dehydrogenase 3 | MT | 0.798 | 0.007 |
| *2610207I05Rik (Smg1)* | ILMN_2930057 | SMG1 homolog, phosphatidylinositol 3-kinase-related kinase (C. elegans) | 7 | 0.798 | 0.009 |
| *D430022H17Rik (Abhd10)* | ILMN_2579504 | abhydrolase domain containing 10, mitochondrial | 16 | 0.797 | 0.019 |
| *Atp5a1* | ILMN_2633229 | ATP synthase, H+ transporting, mitochondrial F1 complex, alpha subunit, isoform 1 | 18 | 0.797 | 0.004 |
| *1810044O22Rik (Cyb5b)* | ILMN_2720180 | cytochrome b5 type B | 8 | 0.797 | 0.005 |
| *Rrm1* | ILMN_1258715 | ribonucleotide reductase M1 | 7 | 0.797 | 0.007 |
| ***Hist1h4h*** | **ILMN_2664129** | **histone cluster 1, H4k** | **13** | **0.796** | **0.039** |
| *4930402E16Rik (Pdpr)* | ILMN_1250521 | pyruvate dehydrogenase phosphatase regulatory subunit | 8 | 0.793 | 0.017 |
| *Lrrc2* | ILMN_3003656 | leucine rich repeat containing 2 | 9 | 0.792 | 0.017 |
| *Hk2* | ILMN_1239397 | Hexokinase 2 | 6 | 0.790 | 0.012 |
| *C330021A05Rik (Dnajc27)* | ILMN_2618831 | DnaJ (Hsp40) homolog, subfamily C, member 27 | 12 | 0.789 | 0.001 |
| *Btg1* | ILMN_2866185 | B-cell translocation gene 1, anti-proliferative; similar to myocardial vascular inhibition factor | 10 | 0.779 | 0.002 |
| *Amy1* | ILMN_2626453 | amylase 1, salivary | 3 | 0.779 | 0.007 |
| *Stard7* | ILMN_2676066 | START domain containing 7 | 2 | 0.775 | 0.0002 |
| *Tmem65* | ILMN_2987564 | transmembrane protein 65 | 15 | 0.773 | 0.010 |
| *Ckmt2* | ILMN_2698052 | creatine kinase, mitochondrial 2 | 13 | 0.766 | 0.011 |
| *2310043N10Rik (Neat1)* | ILMN_2493030 | nuclear paraspeckle assembly transcript 1 (non-protein coding) | 19 | 0.764 | 0.011 |
| *Pnpla2* | ILMN_2687745 | patatin-like phospholipase domain containing 2 | 7 | 0.764 | 0.005 |
| *Trp53inp2* | ILMN_2457585 | transformation related protein 53 inducible nuclear protein 2 | 2 | 0.761 | 0.042 |
| *Dcn* | ILMN_1254458 | decorin | 10 | 0.759 | 0.020 |
| *Ccdc3* | ILMN_2611180 | coiled-coil domain containing 3 | 2 | 0.759 | 0.039 |
| *Dapk2* | ILMN_2977558 | death-associated protein kinase 2 | 9 | 0.757 | 0.024 |
| ***Gpihbp1*** | **ILMN_2614842** | **GPI-anchored HDL-binding protein 1** | **15** | **0.754** | **0.037** |
| *Apoc1* | ILMN_2599794 | apolipoprotein C-I | 7 | 0.753 | 0.017 |
| ***mt-Nd4l*** | **ILMN_2512204** | **mitochondrially encoded NADH dehydrogenase 4L** | **MT** | **0.752** | **0.002** |
| *Serinc3* | ILMN_2787844 | serine incorporator 3 | 2 | 0.746 | 0.043 |
| *Itgb6* | ILMN_2805375 | integrin beta 6 | 2 | 0.734 | 0.044 |
| ***LOC233080 (Ffar3)*** | **ILMN_1236703** | **free fatty acid receptor 3** | **7** | **0.732** | **0.009** |
| *2310003D02Rik (Xirp2)* | ILMN_2497657 | xin actin-binding repeat containing 2 | 2 | 0.722 | 0.01 |
| *Aadacl1 (Nceh1)* | ILMN_2949021 | arylacetamide deacetylase-like 1 | 3 | 0.720 | 0.027 |
| ***Wfs1*** | **ILMN_1224079** | **Wolfram syndrome 1 homolog (human)** | **5** | **0.718** | **0.003** |
| *Mpp3* | ILMN_2662595 | membrane protein, palmitoylated 3 (MAGUK p55 subfamily member 3) | 17 | 0.710 | 0.013 |
| *9630055N22Rik (Mfsd7b)* | ILMN_1222512 | major facilitator superfamily domain containing 7B | 1 | 0.706 | 0.018 |
| *Hp* | ILMN_2944824 | haptoglobin | 8 | 0.690 | 0.013 |
| *Atp1b1* | ILMN_1214703 | non-metastatic cells 7, protein expressed in (nucleoside-diphosphate kinase) | 1 | 0.689 | 0.029 |
| *Itgb6* | ILMN_2805372 | integrin beta 6 | 2 | 0.662 | 0.018 |
| *BC018222* | ILMN_2661366 | transmembrane protein 45b | 9 | 0.648 | 0.00646 |
| *2610528E23Rik* | ILMN_1233531 | RIKEN cDNA 2610528E23 gene | 16 | 0.624 | 0.003 |
| *Mgst1* | ILMN_2940195 | microsomal glutathione S-transferase 1 | 6 | 0.529 | 0.003 |
