# Supplementary material for: Opposite Phenotypes of Muscle Strength and Locomotor Function in Mouse Models of Partial Trisomy and Monosomy 21 for the Proximal Hspa13-App Region
Source: PLoS Genet. 2015 Mar 24;11(3):e1005062. doi: 10.1371/journal.pgen.1005062 (PMC4372517; doi:10.1371/journal.pgen.1005062)
Supplement: S3 Table — Genes whose expression is significantly altered in Ms3Yah gastrocnemius muscles with FC>|1.2| (t-test p<0.05). Gene names from the Hspa13-App region are in italics. Probes deregulated in Ms3Yah muscles are in bold. (DOCX) [file pgen.1005062.s003.docx]

| **Symbol** | **Probe ID** | **Gene name** | **Chromosome location** | **Fold change** | **P value** |
| --- | --- | --- | --- | --- | --- |
| *Angptl7* | ILMN_2844820 | angiopoietin-like 7 | 4 | 2.083 | 0.003 |
| *a* | ILMN_1228049 | nonagouti | 2 | 1.83 | 0.00000003 |
| *Rbp7* | ILMN_2733708 | retinol binding protein 7, cellular | 4 | 1.724 | 0.032 |
| *Angptl7* | ILMN_2844819 | angiopoietin-like 7 | 4 | 1.716 | 0.003 |
| *2900060B14Rik (U4atac)* | ILMN_1253544 | U4atac minor spliceosomal RNA | 1 | 1.713 | 0.045 |
| *2310076L09Rik (Plin5)* | ILMN_3158565 | perilipin 5 | 17 | 1.59 | 0.030 |
| *Sh3kbp1* | ILMN_2755443 | SH3-domain kinase binding protein 1 | X | 1.571 | 0.004 |
| *Col1a1* | ILMN_2687872 | collagen, type I, alpha 1 | 11 | 1.565 | 0.004 |
| *Sparc* | ILMN_3059326 | secreted acidic cysteine rich glycoprotein; similar to Secreted acidic cysteine rich glycoprotein | 11 | 1.506 | 0.002 |
| *Rap1ga1* | ILMN_1227577 | Rap1 GTPase-activating protein | 4 | 1.506 | 0.002 |
| *Acaa2* | ILMN_2704822 | acetyl-Coenzyme A acyltransferase 2 (mitochondrial 3-oxoacyl-Coenzyme A thiolase) | 18 | 1.473 | 0.006 |
| *Acaa2* | ILMN_2704823 | acetyl-Coenzyme A acyltransferase 2 (mitochondrial 3-oxoacyl-Coenzyme A thiolase) | 18 | 1.47 | 0.0007 |
| *Adh1* | ILMN_1258501 | alcohol dehydrogenase 1 (class I) | 3 | 1.468 | 0.0006 |
| *Acaa2* | ILMN_2704826 | acetyl-Coenzyme A acyltransferase 2 (mitochondrial 3-oxoacyl-Coenzyme A thiolase) | 18 | 1.442 | 0.002 |
| *Adh1* | ILMN_2850077 | alcohol dehydrogenase 1 (class I) | 3 | 1.422 | 0.005 |
| *Eno1* | ILMN_2958159 | enolase 1, alpha non-neuron | 4 | 1.404 | 0.012 |
| *As3mt* | ILMN_1247626 | arsenic (+3 oxidation state) methyltransferase | 19 | 1.398 | 0.002 |
| *Actb* | ILMN_2617433 | actin, beta | 5 | 1.388 | 0.016 |
| *scl000408,1_6 (Adk)* | ILMN_2455920 | Adenosine Kinase | 14 | 1.386 | 0.0002 |
| *Sh3kbp1* | ILMN_2881019 | SH3-domain kinase binding protein 1 | X | 1.383 | 0.016 |
| *Knsl5 (Kif23)* | ILMN_1250752 | kinesin family member 23 | 9 | 1.382 | 0.003 |
| *Gbas* | ILMN_2703585 | glioblastoma amplified sequence | 5 | 1.361 | 0.004 |
| *Ramp2* | ILMN_2661422 | receptor (calcitonin) activity modifying protein 2 | 11 | 1.359 | 0.011 |
| *Pcolce* | ILMN_1253741 | procollagen C-endopeptidase enhancer protein | 5 | 1.34 | 0.004 |
| *Lims2* | ILMN_1247916 | LIM and senescent cell antigen like domains 2 | 18 | 1.336 | 0.014 |
| *Acadl* | ILMN_2689473 | acyl-Coenzyme A dehydrogenase, long-chain | 1 | 1.336 | 0.021 |
| *Lims2* | ILMN_2738345 | LIM and senescent cell antigen like domains 2 | 18 | 1.332 | 0.003 |
| *Gys3* | ILMN_2658554 | glycogen synthase 1, muscle | 7 | 1.327 | 0.041 |
| *Nudt7* | ILMN_3123836 | nudix (nucleoside diphosphate linked moiety X)-type motif 7 | 8 | 1.322 | 0.003 |
| *Pank1* | ILMN_2868013 | pantothenate kinase 1 | 19 | 1.315 | 0.002 |
| *Adssl1* | ILMN_2711688 | adenylosuccinate synthetase like 1 | 12 | 1.306 | 0.031 |
| *Adk* | ILMN_2986899 | Adenosine Kinase | 14 | 1.302 | 0.002 |
| *Idh3g* | ILMN_1224368 | isocitrate dehydrogenase 3 (NAD+), gamma | X | 1.302 | 0.001 |
| *LOC433224 ??* | ILMN_3150205 | required for meiotic nuclear division 1 homolog (S. cerevisiae); predicted gene 5512 | 19 | 1.302 | 0.007 |
| *Gja1* | ILMN_1244291 | gap junction protein, alpha 1 | 10 | 1.3 | 0.011 |
| *Acaa2* | ILMN_2788984 | acetyl-Coenzyme A acyltransferase 2 (mitochondrial 3-oxoacyl-Coenzyme A thiolase) | 18 | 1.3 | 0.049 |
| ***Wfs1*** | **ILMN_1224079** | **Wolfram syndrome 1 homolog (human)** | **5** | **1.3** | **0.015** |
| *Mxra8* | ILMN_2759952 | matrix-remodelling associated 8 | 4 | 1.299 | 0.048 |
| *Dffa* | ILMN_2704900 | DNA fragmentation factor, alpha subunit | 4 | 1.292 | 0.007 |
| *Cxcl12* | ILMN_3158250 | chemokine (C-X-C motif) ligand 12 | 6 | 1.291 | 0.018 |
| *2310005E10Rik (Akr1b10)* | ILMN_2733753 | aldo-keto reductase family 1, member B10 (aldose reductase) | 6 | 1.291 | 0.0005 |
| *Alas1* | ILMN_2664040 | aminolevulinic acid synthase 1 | 9 | 1.291 | 0.017 |
| *Gnb5* | ILMN_1237961 | guanine nucleotide binding protein (G protein), beta 5 | 9 | 1.288 | 0.032 |
| *1810054O13Rik* | ILMN_2645662 | transmembrane protein 86A | 7 | 1.287 | 0.008 |
| *Slc25a4* | ILMN_1248759 | solute carrier family 25 (mitochondrial carrier, adenine nucleotide translocator), member 4 | 8 | 1.283 | 0.029 |
| ***LOC233080 (Ffar3)*** | **ILMN_1236703** | **free fatty acid receptor 3** | **7** | **1.28** | **0.008** |
| *0610039N19Rik (Retsat)* | ILMN_1240471 | retinol saturase (all trans retinol 13,14 reductase) | 6 | 1.28 | 0.025 |
| *Nrp* | ILMN_1247094 | neuropilin 1 | 8 | 1.28 | 0.030 |
| *Acadm* | ILMN_1256019 | acyl-Coenzyme A dehydrogenase, medium chain | 3 | 1.278 | 0.006 |
| *Got1* | ILMN_1217043 | glutamate oxaloacetate transaminase 1, soluble | 19 | 1.27 | 0.048 |
| *Prdm2* | ILMN_2707291 | PR domain containing 2, with ZNF domain |  | 1.269 | 0.025 |
| *9130017A15Rik (Pptc7)* | ILMN_2688607 | PTC7 protein phosphatase homolog (S. cerevisiae) | 5 | 1.269 | 0.002 |
| *Mfn2* | ILMN_1259831 | similar to mitofusin 2; mitofusin 2 | 4 | 1.267 | 0.012 |
| *Notch4* | ILMN_1220697 | Notch gene homolog 4 (Drosophila) | 17 | 1.264 | 0.004 |
| ***Hist1h4h*** | **ILMN_2664129** | **histone cluster 1, H4k** | **13** | **1.263** | **0.006** |
| *Gja4* | ILMN_2737704 | gap junction protein, alpha 4 | 4 | 1.258 | 0.028 |
| *Prdm2* | ILMN_1250454 | PR domain containing 2, with ZNF domain |  | 1.258 | 0.025 |
| *Acin1* | ILMN_2755195 | apoptotic chromatin condensation inducer 1 | 14 | 1.255 | 0.011 |
| *Vegfa* | ILMN_2484527 | vascular endothelial growth factor A | 17 | 1.254 | 0.010 |
| *Ppargc1a* | ILMN_2710139 | peroxisome proliferative activated receptor, gamma, coactivator 1 alpha | 5 | 1.254 | 0.003 |
| *P2ry1* | ILMN_2684316 | purinergic receptor P2Y, G-protein coupled 1 | 3 | 1.254 | 0.014 |
| *1110007M04Rik (Ndufaf4)* | ILMN_2734060 | NADH dehydrogenase (ubiquinone) 1 alpha subcomplex, assembly factor 4 | 4 | 1.252 | 0.003 |
| ***Gpihbp1*** | **ILMN_2614842** | **GPI-anchored HDL-binding protein 1** | **15** | **1.251** | **0.019** |
| *Ndufa8* | ILMN_2682019 | NADH dehydrogenase (ubiquinone) 1 alpha subcomplex, 8 | 2 | 1.251 | 0.001 |
| *9530058B02Rik (Fam195a)* | ILMN_2592010 | family with sequence similarity 195, member A | 17 | 1.249 | 0.022 |
| *Car2* | ILMN_2606746 | carbonic anhydrase 2 | 3 | 1.247 | 0.003 |
| *9530058B02Rik (Fam195a)* | ILMN_2904819 | family with sequence similarity 195, member A | 17 | 1.247 | 0.028 |
| *Slc25a20* | ILMN_3138499 | solute carrier family 25 (mitochondrial carnitine/acylcarnitine translocase), member 20 | 9 | 1.246 | 0.021 |
| ***mt-Nd4l*** | **ILMN_2512204** | **mitochondrially encoded NADH dehydrogenase 4L** | **MT** | **1.241** | **0.011** |
| *Coq6* | ILMN_1229589 | coenzyme Q6 homolog (yeast) | 12 | 1.241 | 0.002 |
| *Lims2* | ILMN_2942674 | LIM and senescent cell antigen like domains 2 | 18 | 1.24 | 0.042 |
| *Pitpnc1* | ILMN_2705846 | phosphatidylinositol transfer protein, cytoplasmic 1 | 11 | 1.239 | 0.023 |
| *Vim* | ILMN_2451022 | vimentin | 2 | 1.239 | 0.034 |
| *Snrk* | ILMN_1259356 | SNF related kinase; hypothetical protein LOC100044493; predicted gene 3193 | 9 | 1.238 | 0.0009 |
| *2310005E10Rik (Akr1b10)* | ILMN_2616422 | aldo-keto reductase family 1, member B10 (aldose reductase) | 6 | 1.236 | 0.023 |
| *Pank4* | ILMN_2644140 | pantothenate kinase 4 | 4 | 1.235 | 0.008 |
| *Hmgcl* | ILMN_1256234 | 3-hydroxy-3-methylglutaryl-Coenzyme A lyase | 4 | 1.234 | 0.036 |
| *Cyc1* | ILMN_1251771 | cytochrome c-1 | 15 | 1.234 | 0.0004 |
| *As3mt* | ILMN_2707198 | arsenic (+3 oxidation state) methyltransferase | 19 | 1.231 | 0.002 |
| *Osbpl1a* | ILMN_2577039 | oxysterol binding protein-like 1A | 18 | 1.23 | 0.014 |
| *N6amt1* | ILMN_2668289 | N-6 adenine-specific DNA methyltransferase 1 (putative) | 16 | 1.229 | 0.032 |
| *5830407P18Rik* | ILMN_1237548 |  | 14 | 1.227 | 0.018 |
| *Ank2* | ILMN_2718030 | ankyrin 2, brain | 3 | 1.227 | 0.009 |
| *Slc25a3* | ILMN_2960467 | similar to Solute carrier family 25 (mitochondrial carrier, phosphate carrier), member 3 | 10 | 1.227 | 0.003 |
| *Map3k7ip1* | ILMN_1245918 | mitogen-activated protein kinase kinase kinase 7 interacting protein 1 | 15 | 1.227 | 0.008 |
| *Rmnd1* | ILMN_2955535 | required for meiotic nuclear division 1 homolog (S. cerevisiae); predicted gene 5512 | 10 | 1.226 | 0.028 |
| *Cenpa* | ILMN_1236574 | centromere protein A | 5 | 1.226 | 0.046 |
| *Aifm1* | ILMN_2881263 | apoptosis-inducing factor, mitochondrion-associated 1 | X | 1.225 | 0.006 |
| *Adk* | ILMN_2702471 | Adenosine Kinase | 14 | 1.222 | 0.022 |
| *LOC235480* | ILMN_1221160 | unc-13 homolog C (C. elegans) | 9 | 1.221 | 0.009 |
| *4833424P18Rik (Mrpl47)* | ILMN_2723579 | mitochondrial ribosomal protein L47 | 3 | 1.221 | 0.032 |
| *Brp17 (Pnkd)* | ILMN_2731550 | paroxysmal nonkinesiogenic dyskinesia | 1 | 1.217 | 0.014 |
| *Ppif* | ILMN_3002181 | peptidylprolyl isomerase F (cyclophilin F) | 14 | 1.217 | 0.003 |
| *Slc25a11* | ILMN_2600053 | solute carrier family 25 (mitochondrial carrier oxoglutarate carrier), member 11 | 11 | 1.215 | 0.029 |
| *Coq5* | ILMN_2612738 | coenzyme Q5 homolog, methyltransferase (yeast) | 5 | 1.215 | 0.015 |
| *Tmem143* | ILMN_2608174 | transmembrane protein 143 | 7 | 1.214 | 0.013 |
| *Cxcl12* | ILMN_2737302 | chemokine (C-X-C motif) ligand 12 | 6 | 1.212 | 0.020 |
| *2610027C15Rik (Fam176b)* | ILMN_2890357 | family with sequence similarity 176, member B | 4 | 1.211 | 0.0002 |
| *Actn2* | ILMN_2764727 | actinin alpha 2 | 13 | 1.21 | 0.017 |
| *Sox7* | ILMN_2889482 | SRY-box containing gene 7 | 14 | 1.209 | 0.027 |
| *Hadhb* | ILMN_1230454 | hydroxyacyl-Coenzyme A dehydrogenase/3-ketoacyl-Coenzyme A thiolase/enoyl-Coenzyme A hydratase (trifunctional protein), beta subunit | 5 | 1.208 | 0.009 |
| *2610205H19Rik (Mpc2)* | ILMN_2895991 | brain protein 44; similar to brain protein 44; predicted gene 3982; mitochondrial pyruvate carrier 2 | 1 | 1.206 | 0.029 |
| *2410003P15Rik (Uqcc)* | ILMN_1236994 | ubiquinol-cytochrome c reductase complex chaperone, CBP3 homolog (yeast) | 2 | 1.205 | 0.016 |
| *9430023P16Rik (Smg7)* | ILMN_2682103 | Smg-7 homolog, nonsense mediated mRNA decay factor (C. elegans) | 1 | 0.796 | 0.00001 |
| *Sap30* | ILMN_2694917 | sin3 associated polypeptide | 8 | 0.795 | 0.0009 |
| *Arfgap1* | ILMN_2908546 | ADP-ribosylation factor GTPase activating protein 1 | 2 | 0.792 | 0.003 |
| *Acsl3* | ILMN_3138743 | acyl-CoA synthetase long-chain family member 3 | 1 | 0.791 | 0.031 |
| *Igfbp5* | ILMN_2964324 | insulin-like growth factor binding protein 5 | 1 | 0.784 | 0.025 |
| *Bcl9l* | ILMN_1250469 | B-cell CLL/lymphoma 9-like | 9 | 0.78 | 0.008 |
| *5830404H04Rik (C2cd2)* | ILMN_2944272 | C2 calcium-dependent domain containing 2 | 16 | 0.778 | 0.035 |
| *Jam2* | *ILMN_1227559* | *junction adhesion molecule 2* | *16* | *0.775* | *0.000001* |
| *Atp1b2* | ILMN_2602902 | ATPase, Na+/K+ transporting, beta 2 polypeptide | 11 | 0.769 | 0.0006 |
| *C130023A14Rik* | ILMN_1242271 | RIKEN cDNA C130023A14 gene | 16 | 0.764 | 0.0002 |
| *Ddb1* | ILMN_2620930 | damage specific DNA binding protein 1 | 19 | 0.74 | 0.006 |
| *6430704N06* | ILMN_2472741 | predicted gene 4980 |  | 0.731 | 0.013 |
| *Usp25* | *ILMN_1226508* | *ubiquitin specific peptidase 25* | *16* | *0.729* | *0.002* |
| *2310047C17Rik (Ahnak)* | ILMN_1258578 | AHNAK nucleoprotein (desmoyokin) | 19 | 0.728 | 0.003 |
| ***Atp5j*** | ***ILMN_2632890*** | ***ATP synthase, H+ transporting, mitochondrial F0 complex, subunit F*** | ***16*** | ***0.72*** | ***0.002*** |
| *Hspa1a* | ILMN_2829594 | heat shock protein 1B; heat shock protein 1A; heat shock protein 1-like | 17 | 0.684 | 0.035 |
| *Fzd7* | ILMN_2907560 | frizzled homolog 7 (Drosophila) | 1 | 0.668 | 0.004 |
| *Ddit4l* | ILMN_2695819 | DNA-damage-inducible transcript 4-like | 3 | 0.66 | 0.018 |
| *C920016N10Rik* | ILMN_1252514 |  | 13 | 0.653 | 0.014 |
| ***Atp5j*** | ***ILMN_2962632*** | ***ATP synthase, H+ transporting, mitochondrial F0 complex, subunit F*** | ***16*** | ***0.65*** | ***0.000001*** |
| *Sncg* | ILMN_2939277 | synuclein, gamma | 14 | 0.649 | 0.029 |
| ***D16Ertd472e*** | ***ILMN_1260073*** | ***DNA segment, Chr 16, ERATO Doi 472, expressed*** | ***16*** | ***0.647*** | ***0.0002*** |
| *App* | *ILMN_2597532* | *amyloid beta (A4) precursor protein* | *16* | *0.642* | *0.00007* |
| *B230312E02Rik* | ILMN_2582706 | transducer of ERBB2, 2 |  | 0.64 | 0.010 |
| *Klf2* | ILMN_2604029 | Kruppel-like factor 2 (lung) | 8 | 0.619 | 0.015 |
| ***Chodl*** | ***ILMN_1254082*** | ***chondrolectin*** | ***16*** | ***0.607*** | ***0.0002*** |
| *Adpn (Pnpla3)* | ILMN_2777462 | patatin-like phospholipase domain containing 3 | 15 | 0.604 | 0.032 |
| ***LOC654426*** | ***ILMN_2876629*** | ***ATP synthase, H+ transporting, mitochondrial F0 complex, subunit F pseudogene*** | ***16*** | ***0.601*** | ***0.000005*** |
| *Fos* | ILMN_2750515 | FBJ osteosarcoma oncogene | 12 | 0.591 | 0.048 |
| ***Atp5j*** | ***ILMN_2611261*** | ***ATP synthase, H+ transporting, mitochondrial F0 complex, subunit F*** | ***16*** | ***0.551*** | ***0.000003*** |
| *Sncg* | ILMN_2598478 | synuclein, gamma | 14 | 0.548 | 0.042 |
| *App* | *ILMN_2791028* | *amyloid beta (A4) precursor protein* | *16* | *0.546* | *0.0000006* |
| *Pde4b* | ILMN_2544890 | phosphodiesterase 4B, cAMP specific | 4 | 0.535 | 0.003 |
