# Supplementary figures and images for: Opposite Phenotypes of Muscle Strength and Locomotor Function in Mouse Models of Partial Trisomy and Monosomy 21 for the Proximal Hspa13-App Region
Source: PLoS Genet. 2015 Mar 24;11(3):e1005062. doi: 10.1371/journal.pgen.1005062 (PMC4372517; doi:10.1371/journal.pgen.1005062)

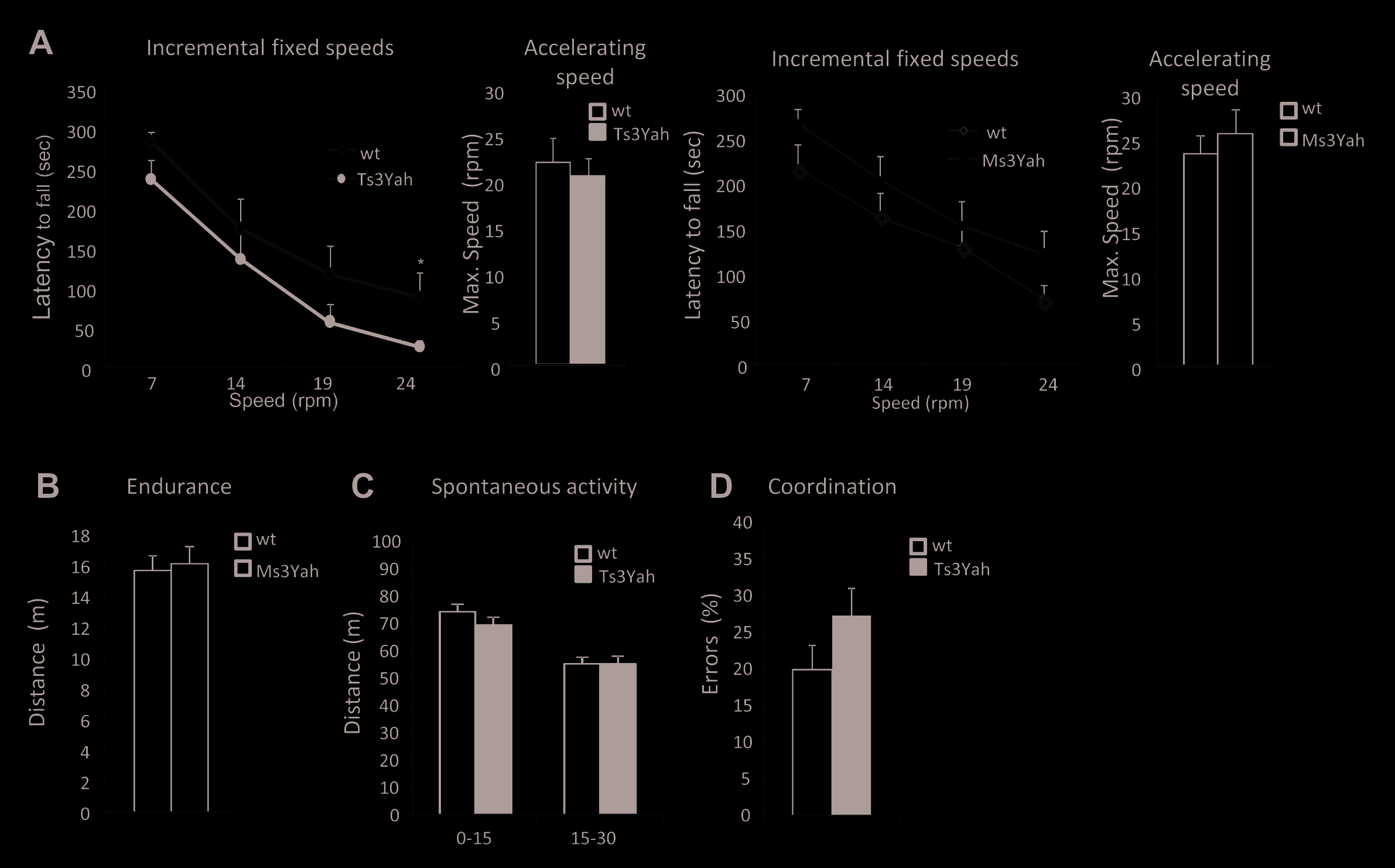

Supplement: S1 Fig — (A) Rotarod tests at incremental fixed speed (repeated ANOVA ‘genotype,’ ‘speed’: Ts3Yah vs diploid: F[1, 69] = 2.395 p = 0.135; Ms3Yah vs diploid: F[1, 90] = 2.607 p = 0.117) and at accelerating speed (two trials [4–40 rpm] in five minutes with the mean rotational velocity at the time of falling calculated for the two trials). (B) Endurance to exercise was measured as the maximal running distance travelled on a treadmill until exhaustion. (C) Spontaneous locomotor activity and habituation were assessed in an open field by measuring the distance travelled during the first 15 minutes and the last 15 minutes of a 30-minute session. (D) Motor coordination skills were tested with a notched bar by looking at the percentage of errors made by the mouse with its hind paws when crossing the notched bar. Data are presented as mean ± sem. (TIF) [file pgen.1005062.s006.tif]

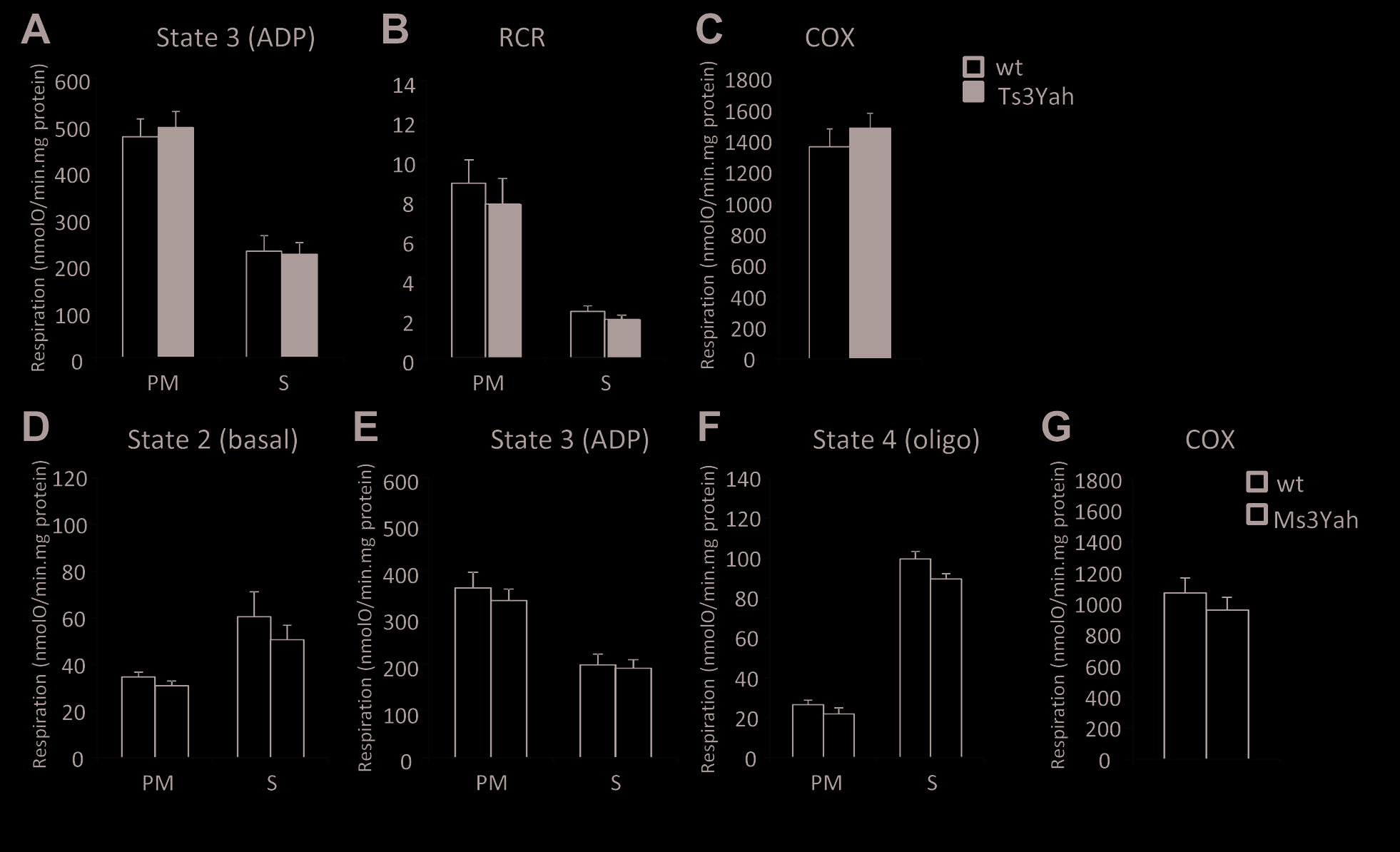

Supplement: S2 Fig — Mitochondria were isolated from hind limb muscle to test their respiration capacities. (A) State 3, ADP-stimulated respiration in Ts3Yah mitochondria. (B) Respiratory control ratio (RCR) is the ratio of state 3 to state 4o. (C) COX activity was monitored by measuring mitochondrial oxygen consumption during PM respiration with the addition of 10 mM antimycin, 2 mM ascorbate, and 0.5 mM TMPD. Yield of mitochondrial protein per gram of muscle is obtained from the mitochondrial extraction. (D–F) Rates of oxygen consumption in Ms3Yah mitochondria measured in the presence of pyruvate/malate (PM) or succinate (S). (D) State 2, basal was measured in the presence of substrates only. (E) State 3, ADP-stimulated respiration (ADP) was measured with the addition of 500 μM ADP. (F) State 4o, after addition of oligomycin. (G) COX activity in Ms3Yah mitochondria. Data are presented as mean ± sem (n = 5–6 per genotype). (TIF) [file pgen.1005062.s007.tif]
