# Supplementary material for: Opposite Phenotypes of Muscle Strength and Locomotor Function in Mouse Models of Partial Trisomy and Monosomy 21 for the Proximal Hspa13-App Region
Source: PLoS Genet. 2015 Mar 24;11(3):e1005062. doi: 10.1371/journal.pgen.1005062 (PMC4372517; doi:10.1371/journal.pgen.1005062)
Supplement: S1 Text — (DOCX) [file pgen.1005062.s008.docx]

# Supporting Information (1764 words)

**Gene targeting and generation of chromosomal rearrangements in ES cells**

The targeting vectors containing DNA fragments that were generated by partial *Sau3A*I digestion of genomic DNA from the 129S5 mouse strain were isolated from the 5’-Hprt and 3’-Hprt libraries [95] and contained loxP sites in the same relative orientation. The genomic inserts (10.2 kb in the 5’-Hprt and 3’-Hprt vectors) are mapped to the *Hspa13* and *App* genes, respectively. Prior to electroporation into HM-1 Hprt-deficient ES cells [96], targeting vectors were linearised in the homology region using the *Afl*II and *Swa*I restriction enzymes, respectively. The 5’-Hprt vector was first inserted, and simple targeted recombinant ES cell clones were selected with G414 and identified by Southern blot analysis. Simple targeted cells were then targeted with the 3’-Hprt, selected with puromycin, and analysed by Southern blot. The selected double-targeted recombinant probes were electroporated with a Cre-expression vector (pHD4-Cre, gift of C. Kellendonk) to induce Cre/loxP-mediated chromosomal rearrangements. The clones carrying the tandem *Hspa13-App* duplication and the reciprocal deletion were selected in the hypoxanthine, aminopterin, and thymidine (HAT) medium and analysed by Southern blot of NdeI-digested ES cell DNA with a probe against the ampicillin gene present in both integration vectors.

**Southern blot analysis**

Genomic tail or ES cell DNA extracts were obtained using the NaCl precipitation technique. About 10 µg of DNA were digested with *Psi*I for Ms3Yah and *Nde*I for Ts3Yah. DNA fragments were separated by electrophoresis on a 0.7% agarose gel and transferred on a nylon membrane. The membrane was then hybridised with a speciﬁc DIG-labelling probe (probe B in Fig 1; wt: 9.3 kb, Ts3Yah: 18.2 kb) (probe D in Fig 1; wt: 12.2 kb, Ms3Yah: 9.9 kb) and revealed with CDP Star (Roche).

**Fluorescent in situ hybridisation**

Interphase nuclei were recovered by ﬁxing a sample of kidney on a slide. Mouse BAC clones located inside (BAC bMQ-34K13) or outside (BAC bMQ-381L17) the *Hspa13-App* region were chosen. Only 1 mg of mouse BAC DNA was used to generate DNA probes labelled DIG-dUTP (bMQ-34K13) and biotin-dUTP (bMQ-381L17) through nick translation. Detection was achieved by the use of both antidigoxigenin-rhodamine and avidin-ﬂuorescein antibodies (Roche, Mannheim, Germany). The slide was mounted with vectashield medium containing DAPI 40, 6 diamidino-2-phenylindole (Vector Laboratories, Burlingame, CA, USA). The images were analysed by SmartCapture2F (Digital Scientiﬁc, Cambridge, UK) using a Zeiss Axioplan 2 microscope equipped with a cooled camera (Photometrics, Tuscon, USA).

**Array-based comparative genomic hybridisation**

To conﬁrm the duplication and the deletion of the *Hspa13-App* region in the Ts3Yah and Ms3Yah models, a gene copy number variation (CNV) study on the whole genome was undertaken using NimbleGen mouse HD2 whole genome CGH oligonucleotide arrays. Comparative analysis was done using DNA extracts from one animal bearing the *Hspa13-App* duplication, one animal bearing the deletion, and one diploid animal that were ﬂuorescently labelled with Cy5 (control) and Cy3 (Ts3Yah and Ms3Yah). After sonication and labelling, DNA is hybridised to the CGH array, followed by washing of the slide according to the manufacturer’s instructions (Roche NimbleGen, Madison, WI, USA). The slides were scanned using a G2565 scanner at 3 µm resolution (Agilent Technologies, Palo Alto, CA, USA), and 200 array images were analysed using the NimbleScan v2.5 software (Roche NimbleGen, Madison, WI, USA) with default parameters incorporating spatial correction. Arrays comprised 2,100,000 isothermal probes 50–75 bp length, with a median spacing of 1.1 kb throughout the genome (UCSC NCBI37/mm9/July 2007 assembly), enabling high-resolution CNV detection between the Ts3Yah, the Ms3Yah, and the diploid control.

**Open field**

The activities of the animals were tested in open boxes (open fields: 42 cm wide, 42 cm large, 40 cm high) made of transparent Plexiglas. Every activity was recorded with an infrared photobeam detection system (Acti-Track, LSI Letica, Panlab). The animals were individually placed in the open field and allowed to move freely for a 30-minute session. The distance they travelled was recorded.

**Rotarod test**

The animals were tested for their ability to maintain balance on a rotating rod of 5 cm diameter (hard plastic covered by a rubber foam). The mice were placed on the rotating rod facing the direction of the rotation. After two training trials of two minutes each at a constant speed of 7 rpm, the mice were tested for their ability to stay on the rotating rod in four consecutive trial sessions with increased fixed rotational speeds (7, 14, 19, and 24 rpm). The animals were allowed to stay on the rod for a maximum of five minutes per trial, with a resting intertrial period of five minutes. After those trial sessions, two tests with accelerating speed from 4 to 40 rpm were performed for five minutes. For each trial, the elapsed time until the mouse fell off the rod was recorded. For the first test at fixed rotational speed, the results were analysed by two-way ANOVA for repeated measures with the genotype as a between-subjects factor and fixed rotational speed as a repeated-measures factor. For the accelerating speed test, a Student’s t-test was used.

**Notched bar test**

This protocol is to test hind limb motor coordination. On the day of the test, in the morning, the mice are ﬁrst taught on a training bar with a large 1.7 mm natural wooden piece and 50 cm long terminal platforms of 6 cm by 6 cm. The training involves successfully crossing the bar 10 times. In the afternoon, the mice are tested on a special notched bar having 12 intermediate raised steps equally distributed between the two platforms. Again, the mice have to cross the bar 10 times. But this time, two observers are placed symmetrically to the bar, looking for hind paw slips outside of the steps and scoring them as errors. Data are given as percentage of errors on total number of crossed steps.

**Grip strength**

The apparatus used for the grip strength test was a stainless steel grid connected to a dynamometer (Force Gauge Lutron). The animal’s four paws were placed on the grid and positioned horizontally and then were gently pulled backwards by the tail in the horizontal plane. The force applied to the grid at the moment the grasp was released was recorded as the peak tension (in g). The test was repeated for three consecutive times within the same session, and the maximum value was taken as the grip strength. Grip strength was expressed in g/body weight.

**SDH on muscle sections**

Tibialis anterioris muscle tissues were collected and immediately frozen in isopentane cooled in liquid nitrogen. There were 10 µm thick serial sections obtained and processed for succinate dehydrogenase (SDH) staining by incubation for one hour at 37°C in a solution of 20 mM potassium dihydrogen phosphate, 80 mM di-sodium hydrogen phosphate, 100 mM sodium succinate, and 2 mg/ml nitro blue tetrazolium. The sections are then fixed in formalin and washed in 15% ethanol before being sealed under a coverslip with an aqueous medium (Dako, S3025).

**Immunofluorescence staining (fibre typing)**

The characterisation of type I fibres was realised by immunostaining using monocolonal antibody against MyHC type I (M8441 from Sigma, dil 1:1000). Frozen sections were fixed for 10 minutes in glacial acetic acid and rinsed in PBS 1X, Triton 0.1% three times for five minutes. Blocking of endogenous avidin/biotin activity and nonspecific antibody interactions was done using the Avidin/Biotin Blocking Kit from Vector (SP-2001), with PBS 1X and 5% normal goat serum. Primary antibody incubation was realised overnight at 4°C, followed by a series of washes with PBS 1X and secondary antibody for one hour at room temperature using the VECTASTAIN Elite ABC Kit IgM PK-4010 from Vector. Signal was amplified with the signal amplification ABC reactive and detected with DAB substrate (diaminobenzidine, Sigma). The sections were mounted using aqueous mounting medium (Dako, S3025).

**Isolation of skeletal muscle mitochondria**

Mitochondria were isolated from the hind limb skeletal muscles of one or two adult mice per mitochondrial preparation. The muscles were weighed, finely minced with scissors, and homogenised with a Potter-Elvehjem homogenizer (five passages) in an ice-cold isolation buffer (10 ml/g tissue) containing 100 mM sucrose, 50 mM KCl, 50 mM Tris, and 5 mM EGTA, pH 7.4. The homogenate was treated with protease (1 mg/g muscle wet weight) and kept on ice for five minutes with occasional stirring. The mixture was diluted 1:2, homogenised with a Potter-Elvehjem homogenizer (three passages), and centrifuged at 1000×g for 10 minutes. The supernatant was centrifuged at 8700×g for 10 minutes. The resulting pellet was suspended in 15 ml isolation buffer and centrifuged at 1000×g for 10 minutes. The supernatant was filtered through cheesecloth and centrifuged at 8700×g for 10 minutes. The resulting pellet was washed once by suspension in the isolation buffer and centrifuged at 8700×g for 10 minutes. The final pellet was suspended in a minimal volume of isolation buffer and kept on ice. The protein concentration of mitochondrial suspensions was determined in duplicate by a biuret method, with bovine serum albumin as the standard.

**Mitochondrial respiration and COX activity**

All assays were performed at 37°C in respiratory buffer containing mitochondria (0.3–0.4 mg protein/ml), 120 mM KCl, 1 mM EGTA, 5 mM KH_2_PO_4_, 2 mM MgCl_2_, 3 mM HEPES, and 0.3% bovine serum albumin, pH 7.4. Oxygen consumption was measured with a Clark oxygen electrode (Rank Brothers Ltd) in a stirred and closed chamber with a volume of 400 µL (n=5). Mitochondrial preparation per genotype at age five months O2 flux for the different states was assessed sequentially as follows: state 2, addition of the respiratory substrate (5 mM pyruvate + 2.5 mM malate [PM] or 5 mM succinate + 5 µM rotenone [S]); state 3, addition of adenosine diphosphate (ADP, 500 µM); and state 4o, addition of 2.5 mg/ml oligomycin. The cytochrome-c oxidase (COX) activity was determined by the mitochondrial respiration rate in the mitochondrial suspension, which includes 10 mM antimycin, 2 mM ascorbate, and 0.5 mM TMPD (N,N,N’,N’-TetraMethyl-p-Phenylene-Diamine). The measurements of COX activity were done in duplicate on n=5 per genotype.

**Mitochondrial membrane potential**

Mitochondria (0.3 mg/ml) were incubated in the respiratory medium supplemented with 5 µM rotenone. The TPMP^+^-sensitive electrode was calibrated with sequential additions up to 2 µM TPMP^+^. Succinate (5 mM) was added to start the reaction in the presence of rotenone (5 µM). Once mitochondria were energised, 500 µM ADP was added, followed by oligomycin (5 µg/mL) and nigericin (60 ng/ml). Nigericin is a potassium/proton antiporter that sets the ΔpH to zero, which allows the whole Δp to be expressed as Δψ. This protocol allows us to calculate the ΔpH value as the difference between membrane potential values in the presence (ΔpH + Δψ) and in the absence (Δψ) of nigericin.
