# Supplementary material for: Opposite Phenotypes of Muscle Strength and Locomotor Function in Mouse Models of Partial Trisomy and Monosomy 21 for the Proximal Hspa13-App Region
Source: PLoS Genet. 2015 Mar 24;11(3):e1005062. doi: 10.1371/journal.pgen.1005062 (PMC4372517; doi:10.1371/journal.pgen.1005062)
Supplement: Supplementary file 6 [file pgen.1005062.s004.docx]

MSigDB Gene set Enrichment in wt vs Ts3Yah mice (down-regulated)	Brief database descrition	Set size	ES	NES	NOM p value	FDR q-val	Leading edge genes
MAP00280_VALINE_LEUCINE_AND_ISOLEUCINE_DEGRADATION	Valine, leucine and isoleucine degradation	26	- 0.851	- 2.576	0.0	0.0	Oxct, Aldh6a1, Ivd, Pccb, Acadm, Hmgcs1, Bckdhb, Hmgcl, Acaa2, Aldh1a1, Bckdha, Hadhb, Mccc1, Aldh3a2
GO_0005739	Mitochondrion	148	-0.606	-2.569	0.0	0.0	Ckmt2, Bdh, Ivd, Pdha1, Pccb, Pdk1, Dld, Acadm, Got2, Bckdhb, Atp5f1, Aco2, Mthfd2, Sod2, Cox5a, Acadvl, Sdha, Cyp27a1, Acaa2, Pdhb, Mipep, Ppox, Bckdha, Dlat, Acp6, Idh2, Hadhb, Bckdk, Cox7a1, Cox15, Coq7, Hadhsc, Ffs1, Tufm, Slc25a3, Shmt2, Etfa, Cox7a2, Hmgcs2, Acadsb, Ucp3, Atp5e, Casp8, Slc25a16, Ndufa8, Afg3l2, Bnip3l, Acads, Acate2, Spg7, Sdhb, Decr1, Cpt1b, Mtrf1, Cyp27b1, Dguok, Crat, Endog, Pdk4, Cox7b, Mrps31, Glud1, Slc25a12, Pmpcb, Arg2, Acadl, Mtif2, Cs, Idh3a, Cyp11b1, Prss15, Tk2
HUMAN_MITODB_6_2002	Mitochondrial genes; based on literature and sequence annotation resources and converted to Affymetrix HG-U133A probe sets.	376	-0.553	-2.554	0.0	0.0	Ckmt2, Ppm2c, Bdh, Ppif, Oxct, Alas1, Ivd, Pdha1, Pccb, Pdk1, Gck, Dld, Acadm, Uqcrc1, Slc1a3, Gcat, Maob, Mfn1, Sucla2, Dlst, Got2, Bckdhb, Prdx3, Atp5f1, Ndufs4, Hmgcl, Slc25a15, Cox10, Tst, Idh3g, Cpt2, Aco2, Ndufs2, Mrpl4, Mthfd2, Sod2, Cox5a, Acadvl, Ogdh, Sdha, Ndufa4, Cyp27a1, Acaa2, Ivns1abp, Pdhb, Got1, Mipep, Ppox, Mrpl18, Bckdha, Mrpl48, Akap1, Ndufa9, Uqcrc2, Dlat, Acp6, Idh2, hadhb, Bckdk, Hspd1, Cox7a1, Cox15, Clpp, Hlcs, Etfdh, Hspa1l, Coq7, Slc25a11, Dmn1l, Hadhsc, Nfs1, Tufm, Slc25a3, Shmt2, Cox6a1, Etfa, Cox7a2, Slc25a10, Mccc1, Cox4i1, Trap1, Aldh4a1, Atp5g1, Mrps18c, Mmgcs2, Acadsb, Cox7c, Uqcr, Ucp3, Surf1, Atp5e, Bcl2, Atp5d, Slc25a20, Nnt, Casp8, Odc1, Timm17b, Mfn2, Mdh2, Mrps7, Mtch1, Slc25a16, Ndufa8, Afg3l2, Bnip3l, Acads, Mrpl9, Sardh, Acate2, Spg7, Frap1, Slc25a1, Hspe1, Bcl2l11, Sdhb, Decr1, Cpt1b, Mtrf1, Ndufb5, Cyp27b1, Cox6c, Dguok, Bnip3, Htatsf1, Ndufc2, Crat, Endog, Mrpl46, Mtch2, Mrpl20, Ndufv2, Mrpl2, Ndufs7, Abcb8, Pdk4, Akap10, Cox7b, Mrps31, Glud1, Mrpl35, Atp5l, Immt, Atp5h, Slc25a12, Pmpcb, Uqcrb, Arg2, Acadl, Tomm70a, Mtif2, Mrps11, Ndufa10, Scp2, Cs
KREBS-TCA_CYCLE	Genes in the Krebs-TCA cycle	26	-0.831	-2.470	0.0	0.0	Ppm2c, Pdha1, Pdk1, Dld, Dlst, Idh3g, Aco2, Ogdh, Sdha, Mdh1, Pdhb, Dlat, Idh2, Pdk2, Mdh2, Sdhb, Pdk4, Cs, Idh3a
MITOCHONDR	Mitochondrial genes	380	-0.5167541	-2.391	0.0	0.0	Ckmt2, Mb, Alas1, Cyp2e1, Ivd, Pdha1, Cabc1, Pccb, Pdk1, Gck, Dld, Acadm, Uqcrc1, Slc1a3, Mrpl3, Maob, Got2, Bckdhb, Prdx3, Atp5f1, Ndufs4, Hmgcl, Slc25a15, Cox10, Tst, Aco2, Ndufs2, Mrpl4, Mthfd2, Sod2, Cox5a, Acadvl, Sdha, Ndufa4, Cyp27a1, Acaa2, Pdhb, Mipep, Ppox, Mrpl18, Bckdha, Mrpl48, Uqcrc2, Dlat, Acp6, Idh2, Hadhb, Bckdk, Hspd1, Cox7a1, Cox15, Prss25, Por, Etfdh, Mto1, Coq7, Slc25a11, Dnm1l, Hadhsc, Nfs1, Tufm, Slc25a3, Snca, Shmt2, Cox6a1, Ywhae, Etfa, Cox7a2, Slc25a10, Cox4i1, Prkcd, Aldh4a1, Atp5g1, Mrps18c, Hmgcs2, Acadsb, Dcnt6, Cox7c, Uqcr, Ucp3, Surf1, Atp5e, Bcl2, Atp5d, Slc25a20, Nnt, Casp8, Timm17b, Mfn2, Mdh2, Mrps7, Mtch1, Slc25a16, Ndufa8, Afg3l2, Bnip3l, Acads, Mrpl9, Sardh, Acate2, Spg7, Slc25a1, Hspe1, Sdhb, Decr1, Cpt1b, Mtrf1, Ndufb5, Cyp27b1, Cox6c, Dguok, Htatsf1, Ndufc2, Crat, Endog, Mrpl46, Mtch2, Ndufv2, Mrpl2, Abcb8, Pdk4, Cox7b, Mrps31, Glud1, Mrpl35, Atp5l, Immt, Atp5h, Pygb, Adck2, Slc25a12, Pmpcb, Uqcrb, Arg2, Cdc42bpb, Acadl, Tomm70a, Map3k1, Mtif2, Mrps11, Ndufa10, Cs, Tomm34, Slc30a9, Slc25a17, Raf1, Idh3a, Cyp11b1, Sirt4, Prss15, Tk2, Stard3
PGC	Genes up-regulated in differentiating C2C12 cells (myoblasts) upon expression of PPARGC1A [GeneID=10891] off an adenoviral vector.	322	-0.528	-2.379	0.0	0.0	Ckmt2, Atp5a1, Atp1b1, Nme7, Rrm1, Stard7, Oxct, Alas1, Tfrc, Pdha1, Sepx1, Ung, Dld, Lrpprc, Glrx, Eno1, Ulk1, Acadm, Uqcrc1, Ptk9l, Esrra, Myh11, Mfn1, Vamp8, Sucla2, Spp1, Prdx3, Mat2b, Idh3g, Aco2, Mapkapk2, Mrpl4, Mthfd2, Col7a1, Sod2, Usp2, S100a1, Ghitm, Sdha, Pdhb, Ptges2, Lpin1, Mrpl18, Akap1, Idh2, Ict1, Hadhb, Map2k1, Hspd1, Cox7a1, Clpp, Pdgfrb, Grsf1, Slc25a11, Fabp3, Nfs1, Tcn2, Cdkn2d, Tufm, Pkm2, Itpr2, Cox6a1, Pfkm, Cox7a2, Apba3, Arih1, Fastk, Pgk1, Rangnrf, Mal, Cox7c, Pcyt2, Nola3, Phlda1, Slc25a20, Mdh2, Ndufa8, Bnip3l, Ptdss2, Sacm1l, Acads, Gss, Frap1, Drpla, Hspe1, Ednra, Nfyc, Slc2a4, Upp1, Prdx2, Smpd1, Sdhb, Lcn2, Bat3, Lppfip2, Ndufb5, Tcirg1, Ruvbl2, Crat, Mrpl46, Mtch2, Abhd5, Mrpl20, Ndufv2, Pdk4, Chrne, Cox7b, Pib5pa, Sf1, Gbas, Atp5l, Fbs1
MAP00020_CITRATE_CYCLE_TCA_CYCLE	Genes involved in Citric acid cycle (TCA cycle)	16	-0.830	-2.246	0.0	0.0	Dld, Idh1, Dlst, Idh3g, Aco2, Ogdh, Sdha, Mdh1, Idh2, Mdh2, Sdhb, Acly, Cs, Idh3a
MAP00650_BUTANOATE_METABOLISM	Butanoate metabolism	21	-0.783	-2.220	0.0	0.0	Bhd, Oxct, Pdha1, Hmgcs1, Hmgcl, Pdhb, Aldh1a1, Hmgcs2, Acads, Aldh3a2, Aldh5a1
ELECTRON_TRANSPORT_CHAIN	Electron transport chain	83	-0.555	-2.125	0.0	0.0	Atp5a1, Uqcrc1, Atp5f1, Ndufs4, Ndufs2, Cox5a, Sdha, Ndufa4, Ndufa9, Uqcrc2, Cox7a1, Cox15, Ndufb9, Cox6a1, Cox7a2, Cox4i1, Atp5g1, Cox7c, Uqcr, Ucp3, Surf1, Atp5e, Atp5d, Ndufa8, Afg3l2, Ndufv1, Sdhb, Ndufb5, Cox6c, Ndufc2, Ndufv2, Ndufs7, Cox7b, Atp5l, Atp5h, Uqcrb, Ndufa10
MAP00071_FATTY_ACID_METABOLISM	Fatty acid metabolism	27	-0.681	-2.076	0.0	2.662E-4	Cyp2e1, Acadm, Cpt2, Acaa2, Aldh1a1, Hadhb, Acads, Adh5, Aldh3a2, Cyp1a1, Acadl, Cyp1a2, Hadha, Cyp4b1
FA		19	-0.759	-2.053	0.0	6.934E-4	Acadm, Cpt2, Acadvl, Acaa2, Hadhb, Hadhsc, Acadsb, Acads, Decr1, Cpt1b, Crat, Acadl
MAP00010_GLYCOLYSIS_GLUCONEOGENESIS	Glycolysis/gluconeogenesis (KEGG)	46	-0.592	-2.016	0.0	0.002	Hk2, Pdha1, Gck, Dld, Eno1, Pdhb, Aldh1a1, Dlat, Fmnl3, Pkm2, Pfkm, Pgk1, Aldh3a1, Adh5, Aldh3a2
MAP00640_PROPANOATE_METABOLISM	Propanoate metabolism (KEGG)	16	-0.758	-2.014	0.0	0.002	Aldh6a1, Pccb, Acacb, Acadm, Aldh1a1, Aldh3a2
FATTY_ACID_DEGRADATION	Fatty acid degradation	19	-0.710	-1.978	0.0	0.003	Acadm, Cpt2, Acadvl, Acaa2, Hadhb, Lpl, Hadhsc, Acadsb,Slc25a20, Acads, Cpt1b, Crat, Acadl
MAP00052_GALACTOSE_METABOLISM	Galactose metabolism (KEGG)	22	-0.667	-1.925	0.0	0.006	Hk2, Gck, Ugp2, Fmnl3, Galt, Pfkm, B4galt2
SPPAPATHWAY	Thrombin cleaves protease-activated receptors PAR1 and PAR4 to induce calcium influx and activate platelet aggregation, a process inhibited by aspirin.	20	-0.678	-1.918	0.0	0.005	Map2k1, Itgb1, F2r, Mapk1, Prkca, Raf1, Pla2g4a, Ptk2, Plcb1, F2rl3
MAP00620_PYRUVATE_METABOLISM	Pyruvate metabolism (KEGG)	24	-0.645	-1.914	0.002	0.006	Pdha1, Acacb, Dld, Mdh1, Pdhb, Aldh1a1, Dlat, Pkm2, Mdh2, Adh5, Aldh3a2
SIG_CHEMOTAXIS	Genes related to chemotaxis	44	-0.558	-1.866	0.0	0.009	Pdk1, Wasl, Cfl2, Inppl1, Mylk, Rock1, Akt3, Itpr2, Pak1, Arhgef11, Gdi1, Pik3cd, Itpr1, Actr2, Btk, Pak4
MAP00120_BILE_ACID_BIOSYNTHESIS	Bile acid biosynthesis (KEGG)	18	-0.695	-1.863	0.0	0.009	Cyp27a1, Acaa2, Aldh1a1, Hadhb, Adh5, Aldh3a2, Srd5a2, Cyp7a1
VOXPHOS	Genes involved in oxidative phosphorylation; based on literature and sequence annotation resources and converted to Affymetrix HG-U133A probe sets.	77	-0.491	-1.851	0.0	0.011	Uqcrc1, Atp5f1, Ndufs4, Cox10, Ndufs2, Cox5a, Sdha, Ndufa4, Ndufa9, Uqcrc2, Cox7a1, Cox15, Cox6a1, Cox7a2, Cox4i1, Atp5g1, Cox7c, Uqcr, Surf1, Atp5e, Atp5d, Ndufa8, Sdhb, Ndufb5, Cox6c, Ndufc2, Ndufs7, Cox7b, Atp5l, Grim19, Atp5h, Uqcrb, Ndufa10
MAP00360_PHENYLALANINE_METABOLISM	Phenylalanine metabolism (KEGG)	15	-0.705	-1.844	0.004	0.012	Maob, Got2, Aoc3, Got1, Aldh3a1
ELECTRON_TRANSPORT		51	-0.532	-1.832	0.0	0.013	Cyp2e1, Uqcrc1, Maob, Cyp27a1, Ppox, Paox, Smox, Cyb561, Surf1, Fads3, Cyp27b1, Cyp1a1, Uqcrcb, Cyp46a1, Alas2, Ptgis, Cyp1a2, Cyp20a1, Cyp7a1, Ern1, Cyp4b1, Eif2ak3, Aof1
MAP00500_STARCH_AND_SUCROSE_METABOLISM	Starch and sucrose metabolism (KEGG)	17	-0.673	-1.798	0.004	0.020	Hk2, Gbe1, Gck, Ugp2, Gusb, Pygb, G6pc
CERAMIDEPATHWAY	Ceramide Signaling Pathway	20	-0.637	-1.785	0.0	0.022	Map2k4, Map2k1, Bcl2, Caps8, Nfkb1, Smpd1, Mapk1, Rela, Map3k1, Raf1, Tnfrsf1a, Fadd, Cycs
MAPK_CASCADE		20	-0.637	-1.770	0.010	0.025	Mbp, Map2k4, Map2k1, Mapk14, Mapk1, Map3k2, Map3k1, Raf1, Map2k7Map2k6
MAPKPATHWAY	MAPKinase Signaling Pathway	84	-0.458	-1.746	0.002	0.032	Rps6ka2, Mapkapk2, Map2k4, Tgfb3, Mapkapk3, Map2k1, Pdzgef1, Rps6ka5, Map4k2, Mknk2, Rps6ka3, Mef2d, Pak1, Mapk14, Map3k8, Nfkb1, Mapk6, Mapk1, Rps6ka4, Map4k3, Rela, Chuk, Map3k2, Mef2a, Mapk11, Map3k1, Raf1, Map2k7, Rac1, Map2k6
