# Supplementary material for: Opposite Phenotypes of Muscle Strength and Locomotor Function in Mouse Models of Partial Trisomy and Monosomy 21 for the Proximal Hspa13-App Region
Source: PLoS Genet. 2015 Mar 24;11(3):e1005062. doi: 10.1371/journal.pgen.1005062 (PMC4372517; doi:10.1371/journal.pgen.1005062)
Supplement: Supplementary file 7 [file pgen.1005062.s005.docx]

MSigDB Gene set Enrichment in Ms3Yah vs wt mice (up-regulated)	Brief database descrition	Set size	ES	NES	NOM p value	FDR q-val	Leading edge genes
HUMAN_MITODB_6_2002	Mitochondrial genes; based on literature and sequence annotation resources and converted to Affymetrix HG-U133A probe sets.	376	0.629	2.880	0.0	0.0	Acaa2, Alas1, Acadl, Got1, Slc25a20, Cyc1, Ppif, Hadhb, Ckmt2, Acad8, Cycs, Mrps27, Mrpl46, Got2, Lars2, Dlat, Acadm, Acadvl, Etfa, Mrpl4, Ndufa9, Ndufa8, Ucp1, Slc25a12, Abcb8, Coq7, Bckdk, Etfdh, Pdhb, Gcdh, Sdhb, Etfb, Idh3a, Cox7a1, Ucp3, Uqcrc1, Mrpl16, Dlst, Mdh2, Cox15, Cox7a2, Cyba, Atp5f1, Bcs1l, Ndufb6, Mrpl15, Mthfd2, Cox5b, Timm44, Fech, Cox10, Cox7b, Dnm1l, Echs1, Atp5j2, Blvra, Bckdha, Ndufs8, Surf1, Ogdh, Dci, Acads, Hmgcl, Nfn1, Atp5c1, Mrps22, Idh3g, Slc1a1, Cpt2, Ndufa10, Sod2, Slc25a5, Pccb, Mrpl2, Ndufb7, Mrps11, Mrps18b, Mrps18a, Stard3, Wars2, Atp5o, Mrpl9, Mrpl17, Mrps7, Idh3b, Atpif1, Mdufs4, Ngfrap1, Dap3, Tufm, Cox4i1, Timm9, Mrps34, Vdac3, Mfn2, Slc25a4, Hccs, Supv3l1, Atp5g1, Mrpl34, Slc25a3, Immt, Vdac2, Bdh, Slc25a11, Decr1, Aco2, Mrps17, Ppm2c, Gck, Mrpl49, Mrps31, Uqcrc2, Mrps28, Shmt1, Ndufs7, Mthfd1, Cox5a, Txnrd2, Ndufa6, Abcb7, Ndufs3, Nnt, Tfam, Tomm22, Ndufb5, Gcat, Hadhsc, Mrpl12, Trap1, Mrpl22, Ndufc2, Akap1, Casp8, Ndufs2, Ndufv2, Sdhd, Glud1, Mrpl44, Prdx3, Diablo, Cox6b, Atp5d, Cry1, Hk1, Ak3, Afg3l2, Mtch2, Tfb1m, Cpo, Mrpl48, Mrpl13, Ivns1abp, Cln3, Uros, Bnip3l, Atp5h, Cox6a1, Shmt2, Pdk4, Cpt1b, Cox11, Mipep, Oat, Ndufa5, Atp5g2, Cox7c, Cs, Gls, Opa1, Pycs
MITOCHONDR	Mitochondrial genes	380	0.620	2.854	0.0	0.0	Acaa2, Alas1, Acadl, Slc25a20, Cyc1, Hadhb, Ckmt2, Acad8, Cycs, Mrps27, Mrpl46, Got2, Lars2, Dlat, Acadm, Acadvl, Etfa, Mrpl4, Ndufa8, Pla2g1b, Ucp1, Slc25a12, Abcb8, Coq7, Bckdk, Etfdh, Pdhb, Gcdh, Sdhb, Etfb, Idh3a, Cox7a1, Ucp3, Uqcrc1, Gnas, Mrpl16, Mdh2, Cox15, Cox7a2, Cyba, Atp5f1, Bcs1l, Cabc1, Ndufb6, Mrpl15, Mthfd2, Cox5b, Timm44, Fech, Mrpl3, Cox10, Cox7b, Dnm1l, Echs1, Atp5j2, Bckdha, C1qbp, Ndufs8, Surf1, Dci, Acads, Hmgcl, Atp5c1, Mrps22, Slc1a1, Ndufa10, Sod2, Slc25a5, Pccb, Mrpl2, Ndufb7, Mrps11, Mrps18b, Mrps18a, Stard3, Wars2, Atp5o, Nme2, Mrpl9, Mrpl17, Mrps7, idh3b, Atpif1, Ndufs4, Ngfrap1, Dap3, Tufm, Cox4i1, Timm9, Mrps34, Vdac3, Mfn2, Slc25a4, Hccs, Supv3l1, Atp5g1, Mrpl34, Nt5m, Slc25a3, Immt, Vdac2, Bdh, Slc25a11, Lgals3, Decr1, Aco2, Mrps17, Gck, Mrpl49, Mrps31, Peo1, Uqcrc2, Mrps28, Tfb2m, Mthfd1, Cox5a, Txnrd2, Ndufa6, Mtx1, Abcb7, Ndufs3, Nnt, Tfam, Tomm22, Ndufb5, Hadhsc, Mrpl12, Prss25, Mrpl22, Ndufc2, Sirt4, Casp8, Ndufs2, Ndufv2, Sdhd, Glud1, Mrpl44, Prdx3, Diablo, Cox6b, Peci, Atp5d, Cry1, Hk1, Ak3, Afg3l2, Mtch2, Tfb1m, Cpo, Mrpl48, Mrpl13, Cln3, Bnip3l, Atp5h, Cox6a1, Shmt2, Pdk4, Cpt1b, Cox11, Mipep, Oat, Ndufa5, Atp5g2, Cox7c, Cs
ELECTRON_TRANSPORT_CHAIN	Electron transport chain	83	0.731	2.758	0.0	0.0	Cycs, Ndufa9, Ndufa8, Ucp1, Sdhb, Cox7a1, Ucp3, Uqcrc1, Ndufv1, Cox15, Cox7a2, Atp5f1, Ndufb6, Cox5b, Ndufb9, Cox7b, Atp5j2, Ndufs8, Surf1, Atp5c1, Ndufa10, Slc25a5, Ndufb7, Atp5o, Atpif1, Ndufs4, Cox4i1, Slc25a4, Atp5g1, Ndufb10, Uqcrc2, Ndufs7, Cox5a, Ndufa6, Ndufs3, Ndufb5, Ndufc2, Atp5a1, Ndufs2, Ndufv2, Sdhd, Cox6b, Atp5d, Afg3l2, Atp5h, Cox6a1, Cox11, Sco1, Ndufa5, Atp5g2, Cox7c
GO_0005739 (mitochondrion)	Mitochondrial genes	148	0.657	2.715	0.0	0.0	Acaa2, acadl, Cyc1, Hadhb, Ckmt2, Cycs, Got2, Dlat, Acadm, Acadvl, Etfa, Ndufa8, Ucp1, Slc25a12, Coq7, Bckdk, Pdhb, Gcdh, Sdhb, Idh3a, Cox7a1, Ucp3, Cox15, Cox7a2, Cyba, Atp5f1, Mthfd2, Cox7b, Echs1, Bckdha, Dci, Acads, Atp5c1, Sod2, Pccb, Wars2, Idh3b, Atpif1, Ngfrap1, Tufm, Slc25a4, Supv3l1, Nt5m, Slc25a3, Bdh, Decr1, Aco2, Mrps31, Mthfd1, Cox5a, Txnrd2, Hadhsc, Casp8, Sdhd, Glud1, Diablo, Cry1, Afg3l2, Cpo, Cln3, Bnip3l, Shmt2, Pdk4, Cpt1b, Cox11, Mipep, Oat, Cs
PGC	Genes up-regulated in differentiating C2C12 cells (myoblasts) upon expression of PPARGC1A [GeneID=10891] off an adenoviral vector.	322	0.565	2.573	0.0	0.0	Eno1, Gbas, Pcolce, Dffa, Alas1, Acadl, Tfrc, Slc25a20, Cyc1, Atp1b1, Nme7, Osbpl1a, Fabp3, Hadhb, Ckmt2, Cycs, Mrpl46, Mpp5, Acadm, Mrpl4, Ndufa8, Esrra, Pdhb, Ung, Gcdh, Sdhb, Etfb, Idh3a, Cox7a1, Psca, Pfkp; Muc5ac, Polr2e, Uqcrc1, Mdh2, Cox7a2, Ndufb6, Mrpl15, Mthfd2, Cox5b, Pmm1, Timm44, Grsf1, Adsl, Cox7b, Rangnrf, Usp2, Chrne, Acads, Mfn1, Atp5c1, Idh3g, Sod2, Slc25a5, Pgls, Mrps18b, Dusp6, Mrps18a, Atp5o, Mapk8, Rnf5, Kcnn1, Ngfrap1, Tufm, Dpysl3, Ptdsr, Mrps34, Vdac3, Slc25a4, Hccs, Supv3l1, Atp1a1, Itpk1, Mrtpl34, Glrx, Comt, P4ha2, Nfyc, Itpr2, Slc25a11, Them2, Aco2, Mrps17, Gsto1, Ptges2, Tfb2m, Shmt1, Rpa3, Ndufa6, Mtx1, Ndufs3, Ndufb5, Lrpprc, Akap1, Atp5a1, Upp1, Ndufv2, Prdx3, Grpel1, Ncam1, Ict1, Mtch2, Mrpl13, Prdx2, Inhba, Mal, Dnaja3, Ednra, Ghitm, Rrm1, Bnip3l, Ptprd, Hspa5, Guk1, Cox6a1, Cx3cl1, Psmb5, Sepx1, Pdk4, Ndufa5, Cox7c, Cs, Lrrfip2, Pgm1
VOXPHOS	Genes involved in oxidative phosphorylation; based on literature and sequence annotation resources and converted to Affymetrix HG-U133A probe sets.	77	0.68966365	2.510	0.0	0.0	Cyc1, Cycs, Ndufa9, Ndufa8, Sdhb, Cox7a1, Uqcrc1, Cox15, Cox7a2, Atp5f1, Bcs1l, Ndufb6, Cox5b, Cox10, Cox7b, Atp5j2, Ndufs8, Surf1, Atp5c1, Ndufa10, Ndufb7, Atp5o? Ndufs4, Ngfrap1, CoxAi1, Atp5g1, Uqcrc2, Ndufs7, Cox5a, Ndufa6, Ndufs3, Ndufb5, Ndufc2, Ndufs2, Sdhd, Cox6b, Atp5d, Atp5h, Cox6a1, Cox11, Ndufa5, Atp5g2, Cox7c
FA	Fatty acid	19	0.825	2.313	0.0	0.0	Acaa2, Acadl, hadhb, Acadm, Acadvl, Echs1, Dci, Acads, Cpt2, Decr1, Hadhsc
MAP00020_CITRATE_CYCLE_TCA_CYCLE	Genes involved in Citric acid cycle (TCA cycle)	16	0.819	2.190	0.0	8.635E-5	Mdh1, Sdhb, Idh3a, Dlst, Mdh2, Ogdh, Idh3g, Aco2, Acly
FATTY_ACID_DEGRADATION	Fatty-acid degradation	19	0.784	2.189	0.0	7.675E-5	Acaa2, Acadl, Slc25a20, Hadhb, Acadm, Acadvl, Acads, Cpt2, Lpl, Hadhsc
KREBS-TCA_CYCLE	Genes in the Krebs-TCA cycle	26	0.733	2.167	0.0	6.908E-5	Mdh1, Dlat, Pdhb, Sdhb, Idh3a, Dlst, Mdh2, Ogdh, Idh3g, Idh3b, Aco2, Ppm2c, Sdhd, Pdk4, Cs
MAP00280_VALINE_LEUCINE_AND_ISOLEUCINE_DEGRADATION	Valine, leucine and isoleucine degradation	26	0.697	2.099	0.0	1.386E-4	Acaa2, Hadhb, Acadm, Echs1, Bckdha, Acads, Hmgcl, Pccb
MAP00071_FATTY_ACID_METABOLISM	Fatty-acid metabolism (KEGG)	27	0.704	2.079	0.0	2.465E-4	Acaa2, Acadl, Hadhb, Acadm, Gcdh, Echs1, Acads, Cpt2, Adh7
MAP00650_BUTANOATE_METABOLISM	Butanoate metabolism (KEGG)	21	0.667	1.891	0.002	0.008	Pdhb, Echs1, Acads, Hmgcl, Bdh, Aldh3a2, Aldh5a1, Abat, Hmgccs2, Oxct
MAP00190_OXIDATIVE_PHOSPHORYLATION	Oxidative phosphorylation (KEGG)	43	0.567	1.888	0.0	0.008	Mpp5, Atp4b, Sdhb, Cox7a1, Uqcrc1, Cox7a2, Cox5b, Cox7b, Cox4i1, Uqcrc2, Cox5a, Atp6v1g1, Ndufv2, Cox6b, Cox6a1, Atp6v0e, Ndufa5, Cox7c
MAP00051_FRUCTOSE_AND_MANNOSE_METABOLISM	Fructose and mannose metabolism	22	0.659	1.875	0.002	0.008	Fmnl3, Pfkp, Pmm1, Fbp2, Pmm2, Gck, Hk1, Hk3, Khk, Pfkl, Gmppa, Grcc9, Fbp1
KRAS_TOP100_KNOCKDOWN	Genes uptergulated in kras knockdown vs controls in a human cell line	74	0.494	1.811	0.0	0.016	Col1a1, Col4a2, Bmp1, Tns, Gadd45b, Sparc, Wisp2, Col5a1, Inpp4b, Col4a1, Pgls, Igfbp7, Evl, P4ha2, Tgfbi, Hspg2, Chst3, St5, Serpine2, Waspip, Cldn4, Adcy7, Slc22a5
MAP00010_GLYCOLYSIS_GLUCONEOGENESIS	Glycolysis and gluconeogenesis	46	0.531	1.787	0.002	0.020	Eno1, Fmnl3, Dlat, Pdhb, Pfkp, Fbp2, Adh7, Gck, Aldh3a2, Hk1, Pgm1, Hk3, Pgam2, Adh5, Aldh3a1, Aldh3b1, Pfkl, Adh4, Grcc9, Fbp1, Acyp2, Dld, Aldoa
INFLAMMATORY_RESPONSE_PATHWAY	Inflammatory response pathway	25	0.602	1.783	0.002	0.020	Col1a1, Fn1, Vtn, Col1a2, Col3a1, Thbs3
MAP00310_LYSINE_DEGRADATION	Lysine degradation (KEGG)	15	0.694	1.759	0.006	0.025	Gcdh, Dlst, Echs1, Ogdh, Shmt2, Aldh3a2, Shmt2
GLUCO		23	0.604	1.731	0.006	0.031	Eno1, Mdh1, Mdh2, Fbp2
EMT_UP	genes up-regulated for epithelial plasticity in tumor progression	55	0.490	1.718	0.0	0.034	Pcolce, Vim, Ppic, Sparc, Dcn, Tcn, Mmp2, Mthfd2, Mmp12, Pmp22, Col6a1, Cd68, Col6a2, Sdc2, Upp1, Inhba
MAP00120_BILE_ACID_BIOSYNTHESIS	Bile acid biosynthesis (KEGG)	18	0.629	1.689	0.006	0.043	Acaa2, Hadhb, Adh7, Aldh3a2, Adh5, Soat1, Adh4, Acaa1
